# Correction: Energy-Optimal Electrical-Stimulation Pulses Shaped by the Least-Action Principle

**DOI:** 10.1371/journal.pone.0124704

**Published:** 2015-04-10

**Authors:** The PLOS ONE Staff

There is an error in Equation 18. Please view the complete, correct equation here:

$$\Theta \left(x\left(T\right)\right)=\frac{K_{penalty}}{2}{\left(V_{THR}-V\left(T\right)\right)}^{2}$$

There is an error in Equation 19. Please view the complete, correct equation here:

$$\begin{array}{l} L=\Theta (x(T))+\int_{t=0}^{T}\left[f_{0}(X,u)-\lambda '(\frac{d}{dt}X-F(X,u))\right]dt\\ \;=\Theta (x(T))+{\left[\lambda 'X\right]}_{t=0}^{T}+\int_{t=0}^{T}\left[H+\frac{d}{dt}\lambda 'X\right]dt \end{array}$$

## References

[1] Krouchev NI, Danner SM, Vinet A, Rattay F, Sawan M (2014) Energy-Optimal Electrical-Stimulation Pulses Shaped by the Least-Action Principle. PLoS ONE 9(3): e90480 doi: 10.1371/journal.pone.0090480 24625822 10.1371/journal.pone.0090480 PMC3953645

