# Supplementary material for: Correction: Energy-Optimal Electrical-Stimulation Pulses Shaped by the Least-Action Principle
Source: PLoS One. 2014 Dec 12;9(12):e115928. doi: 10.1371/journal.pone.0115928 (PMC4264884; doi:10.1371/journal.pone.0115928)
Supplement: File S2 — Republished corrected article. (PDF) [file pone.0115928.s002.pdf]

Energy-Optimal Electrical-Stimulation Pulses Shaped by the Least-Action Principle

Nedialko I. Krouchev[1]*, Simon M. Danner[2,3], Alain Vinet[4], Frank Rattay[2], Mohamad Sawan[1]

1 Polystim Neurotechnologies, Ecole Polytechnique, Montreal, Quebec, Canada, **2** Institute for Analysis and Scientific Computing, University of Technology, Vienna, Austria, **3** Center for Medical Physics and Biomedical Engineering, Medical University, Vienna, Austria, **4** Institut de Genie Biomedical, Universite de Montreal, Montreal, Quebec, Canada

Abstract

Electrical stimulation (ES) devices interact with excitable neural tissue toward eliciting action potentials (AP's) by specific current patterns. Low-energy ES prevents tissue damage and loss of specificity. Hence to identify optimal stimulation-current waveforms is a relevant problem, whose solution may have significant impact on the related medical (e.g. minimized side-effects) and engineering (e.g. maximized battery-life) efficiency. This has typically been addressed by simulation (of a given excitable-tissue model) and iterative numerical optimization with *hard* discontinuous constraints - e.g. AP's are all-or-none phenomena. Such approach is computationally expensive, while the solution is uncertain - e.g. may converge to local-only energy-minima and be model-specific. We exploit the *Least-Action Principle* (LAP). First, we derive in *closed form* the general *template* of the membrane-potential's temporal trajectory, which minimizes the ES energy integral over time and over *any* space-clamp ionic current model. From the *given* model we then obtain the *specific* energy-efficient current waveform, which is demonstrated to be *globally optimal*. The solution is model-independent by construction. We illustrate the approach by a broad set of example situations with some of the most popular ionic current models from the literature. The proposed approach may result in the significant improvement of solution efficiency: cumbersome and uncertain iteration is replaced by a single quadrature of a system of ordinary differential equations. The approach is further validated by enabling a general comparison to the conventional simulation and optimization results from the literature, including one of our own, based on finite-horizon optimal control. Applying the LAP also resulted in a number of general ES optimality principles. One such succinct observation is that ES with long pulse durations is much more sensitive to the pulse's shape whereas a rectangular pulse is most frequently optimal for short pulse durations.

Citation: Krouchev NI, Danner SM, Vinet A, Rattay F, Sawan M (2014) Energy-Optimal Electrical-Stimulation Pulses Shaped by the Least-Action Principle. PLoS ONE 9(3): e90480. doi:10.1371/journal.pone.0090480

Editor: Dante R. Chialvo, National Research & Technology Council, Argentina

Received November 11, 2013; **Accepted** January 30, 2014; **Published** March 13, 2014

Copyright: © 2014 Krouchev et al. This is an open-access article distributed under the terms of the Creative Commons Attribution License, which permits unrestricted use, distribution, and reproduction in any medium, provided the original author and source are credited.

Funding: The Fonds de recherche du Quebec - Nature et technologies and the Natural Sciences and Engineering Research Council of Canada provided funding for this work. S.D. was supported by the Vienna Science and Technology Fund (WWTF), Proj.Nr. LS11-057. The funders had no role in study design, data collection and analysis, decision to publish, or preparation of the manuscript.

Competing Interests: The authors have declared that no competing interests exist.

* Email: Nedialko.Krouchev@polymtl.ca

Introduction

Electrical stimulation (ES) today is an industry worth in excess of 3 G$. ES devices interact with living tissues toward repairing, restoring or substituting normal sensory or motor function [1]. The rehabilitation-engineering applications scope is constantly growing: from intelligent limb prosthetics and deep-brain stimulation (DBS) to bi-directional brain-machine interfaces (BMI), which are no longer *just* about recording brain activity, but have also recently used ES toward *closed-loop* systems, [2–5].

Application-specific current patterns need to be injected toward reliably eliciting *action potentials* (AP's) in target excitable neural tissue. To prevent tissue damage or loss of functional specificity, the employed current waveforms need to be *efficient*. This may significantly impact the biomedical effects and engineering feasibility. Hence, an optimization problem of high relevance to the design of viable ES devices is to minimize the energy required by the stimulation waveforms, while maintaining their capacity for AP triggering toward achieving the targeted functional effects.

A number of recent studies of ES optimality are based on extensive model simulation and related numerical methods through the wider spread of high-performance computing, e.g. [6–9]. The model dynamics to iterate can be arbitrarily complex and nonlinear. This implies lengthy numerically-intensive computation, irregular convergence and constraints that may be difficult to enforce - e.g. that an AP is an *all-or-none* phenomenon. Thus, any function of membrane voltage will suffer dramatic discontinuities at parameter-space manifold boundaries where intermittent AP's are likely to be elicited.

Hence, such an iterative approach is not only computationally expensive, but its solution quality is highly uncertain and model-specific. The long-lasting iteration may converge to shallow local energy-minima. Such numerical misdemeanor of the approach is well known to its frequent users.

In this work we follow the ES pioneers - we use physical reasoning and related mathematics toward a more theoretical treatment of the subject.

Below we summarize very briefly our historical premises. ES' theoretical cornerstones were laid a century ago by experimentally-driven assumptions and models, [10–12]. Various constant ES current levels and durations were tried systematically. E.g. Louis and Marcelle Lapicque spent many years performing such lab

experiments with multiple physiological preparations [13,14]. This classical work led to concepts like *strength-duration curve (SD)*, i.e. the function of threshold (but still AP-evoking) ES current strength on duration. The first mathematical fit to this empirical results is usually attributed to Weiss, [10,15]

$$I_{THR}(T) = b(1 + c/T) \tag{1}$$

where T is the stimulus duration, b is called the *rheobase* (or rheobasic current level) and c is the *chronaxie*.

The most expedite way of introducing the *rheobase* and *chronaxie* would be to point to eqn. (1) and notice that:

$$\lim_{T \to \infty} I_{THR}(T) = b \tag{2}$$

and

$$I_{THR}(c) = 2b \tag{3}$$

i.e. the rheobase is the threshold current strength with very long duration, and chronaxie is the duration with twice the rheobasic current level. In the pioneering studies electrical stimulation was done with extracellular electrodes.

Eqn. (1) is the most simplistic of the 2 'simple' mathematical descriptors of the dependence of current strength on duration, and leads to Weiss' linear charge-transfer progression with T, $Q(T) = T \times I_{THR} = b \times (T + c)$. Both Lapicque's own writings - [11–13], and more recent work are at odds with the linear-charge approximation. Already in 1907 Lapicque was using a linear first-order approximation of the cell membrane, modeled as a single-RC equivalent circuit with fixed threshold:

$$I_{THR}(T) = \frac{b}{1 - \mathbf{e}^{-T/\tau}} = b + \frac{b e^{-T/\tau}}{1 - \mathbf{e}^{-T/\tau}} \tag{4}$$

with time constant $\tau = C/g$; C and $g = 1/R$ are the membrane capacity and conductance respectively.

The second form of eqn. (4) is easily obtained by subtracting/adding the term $be^{-T/\tau}$. From it, when $\tau \gg T$ (and hence $\mathbf{e}^{-T/\tau} \to 1$):

$$I_{THR}(T) \approx b(1 + \tau/T)$$

which accounts for the hyperbolic shape of the classic Lapicque SD curve.

Originally, eqn. (4) described the SD relationship for extra-cellular applied current. However, the single-RC equivalent circuit with fixed threshold, where I is the electrode current flowing across the cell membrane

$$C\dot{v} + v/R = I \tag{5}$$

can be used with either extra- or intra-cellular stimulation. $v = (V - V_{rest})$ is the *reduced* membrane voltage with V_{rest} the *resting* value of V. From eqns. (4) and (5), one may also see that $b = g(V_{THR} - V_{rest})$, where V_{THR} is the attained membrane voltage at the end of the stimulation (at time T).

Notice that the chronaxie c is not explicitly present in eqn. (4). Notice also that - with very short duration $T \ll \tau$, by the Taylor series decomposition of the exponent (around $T = 0$), one may have either $I_{THR}(T) \approx b\tau/T$ or $I_{THR}(T) = b[1 + \tau/T]$. Note that these two different simplifications (and esp. the latter) are 'historical' and depend on which of the two right-hand sides (RHS') of eqn. (4) is used. In the second case only the denominator is developed to first order, while the numerator is truncated at zero-order. The second approximation throws a bridge to Weiss' empirical formula of eqn. (1). I.e. the latter is a simplification of a simplification (i.e. of the 1st-order linear membrane model), capturing best the cases of shortest duration. On the other hand, $I_{THR}(T) \approx b\tau/T$ leads to a constant-charge approximation. Interestingly, the latter may fit well also more complex models of the excitable membrane, which take into account ion-channel gating mechanisms, as well as intracellular current flow, which may be the main contributors for deviations from both simple formulas. These 'subtleties' are all clearly described in Lapicque's work, but less clearly by one of the most recent accounts in [16].

Before we continue, it is in order to examine the practical value of numerical optimization to identify energy-efficient waveforms. It is limited for the following reasons. First, it is subject to the rigorous constraints of *quantitative* equivalence between the model used and the real preparation to which the results should apply. A noteworthy example is provided by the very practice of numerical simulations: often a minute change in parameters precludes the use of a *just computed* waveform, which is no longer able to elicit an AP in the targeted excitable model. Alas, the same or similar applies hundredfold to the real ES practice.

Second, in the search for minimum-energy waveforms, using numerical mathematical programming algorithms, there is no guarantee about obtaining a globally optimal solution.

Finally, such an approach sheds very little light with respect to the major forces that are at play, and the key factors which determine excitability, such as - for example, the threshold value of membrane potential, whose crossing triggers an AP.

However, the problem at hand is also reminiscent of the search for energy-efficiency in many other physical domains - e.g. ecological car driving. For centuries, physics has tackled similar problems through an approach known as *the Least-Action Principle* (LAP) [17].

Thus, we first used simple models to derive key analytical results. We then identified generally applicable optimality principles. Finally, we demonstrate how these principles apply also to far more complex and realistic models and their simulations.

The modeling and algorithmic part of this work is laid out in the next section. First, we introduce a simple and general model *template*. Next we present four most popular specific ionic-current models. Each of these can be *plugged* in the template to describe an ES target in a single spatial location in excitable-tissue (or alternatively - a space-clamped neural process).

We then examine the conditions for the existence of a *finite* membrane-voltage threshold for AP initiation. The introduced ionic-current model properties are analyzed to gain important insights into the solution of the main problem at hand.

Two very different ways to identify energy-efficient waveforms are presented in the last two subsections of the Methods. The first relies on a *standard* numerical optimal-control (OC) approach. The second outlines the LAP in its ES form, which is used to derive a general analytic solution for the energy-optimal trajectories in time of the membrane-potential and stimulation-current.

The Results section presents the model-specific results, applying OC or the LAP. We perform a detailed optimality analysis for both the simple and more realistic models. Comparisons between the two types of approaches, and the quality of their solutions, are made.

Commonly used abbreviations are summarized in Table 1 and symbols - in Table 2.

Table 1. Commonly used abbreviations.

Symbol	Description
0D	zero-dimensional, i.e. single-compartment or space clamp models; whose spatial extents are confined to a point
1D	cable-like, multi-compartment spatial structure; homo-morphic to line
2D etc.	two- or more dimensional, refers to the number of states that describe the excitable system's dynamics
AIS	the axon's initial segment
AP	Action potential
ASA	Adjoint Sensitivity Analysis
BCI	brain-computer interface
BMI	brain-machine interface
BVP	Boundary-value [ODE solution] problem
BVDP	the Bonhoeffer-Van der Pol oscillator-dynamics model; also known as the Fitzhugh-Nagumo model
DBS	Deep-brain stimulation
ES	Electrical stimulation
FHOC	Finite-Horizon Optimal-Control
FP	Fixed point of system dynamics $\rightarrow$ vanishing derivative(s)
HH or HHM	Hodgkin and Huxley's [model of excitable membranes]
IM	the Izhikevich model
LM	the Linear sub-threshold model; also known in computational neuroscience as leaky integrate & fire
MRG	the McIntyre, Richardson, and Grill model
OC	Optimal-Control
ODE	Ordinary Differential equation; see also PDE
PDE	Differential equation involving partial derivatives; see also ODE
LAP	the Least-Action Principle
RN	Ranvier-node
RHS	right-hand side
SD	*strength-duration* [curve]
W.R.T.	with respect to

doi:10.1371/journal.pone.0090480.t001

Table 2. Commonly used symbols.

Symbol	Description
C or C_m	membrane capacity
Δt	the temporal precision of a model's simulation
g or g_m	membrane conductance; see also R_m
g_X	nominal (max.) conductance for ion X
GE	the growing-exponent stimulation pulse
I_S	stimulation current, see also $u(t)$
I_C	the capacitive current, see also C_m
$I_{THR}(T)$	threshold current for duration T to elicit an AP; see T_{STIM}
I_{axial}	algebraic sum of in and out axial currents
$I_{ion}(V(t))$	ionic current function of membrane voltage; see $V(t)$
$I_{ion,0}(V)$	*resting*-state approximation; see x_0
$I_{ion,\infty}(V)$	*asymptotic*-state approximation; see $x_\infty(V)$
λ	cable spatial constant
R or R_m	membrane resistance; see also g_m
P and $P(T)$	power for $u(t)$ as function of duration; see $u(t)$, T_{STIM}
Q and $Q(T)$	charge-transfer
SQR	square (rectangular) waveform
T_{CR}	critical duration; see T_{STIM}
T_{STIM} or T_S or T	duration of stimulation
τ or τ_m	membrane time constant
τ_{ion} or τ_X	gate time constant for ion X
$u(t)$	stimulation waveform
$u^*(t)$	optimal current stimulation waveform
V	membrane voltage
V_r or V_{rest}	*resting* V
$v = V - V_R$	voltage difference w.r.t. rest
$\dot{V}$ or dV/dt	first time-derivative of the membrane voltage
$V(t)$	temporal pattern of V
$V^*(t)$	optimal $V(t)$
V_{THR}	AP triggering V threshold
$V_{THR,0}$	*resting*-state V_{THR}
$V_{THR,\infty}$	the *asymptotic*-state V_{THR}
$x_0 = x_\infty(V_r)$	gate *resting* state for ion X; see V_r
$x_\infty(V) = \lim_{t\to\infty} x(t\|V)$	gate *asymptotic* state for ion X

doi:10.1371/journal.pone.0090480.t002

Methods

A General Excitability Model Template

For the equivalent circuit of Fig. 1, I_S is the stimulation current. I_C is the capacitive current, whose direction is as shown on the Figure when the excitable-membrane's potential is being *depolarized*. The algebraic sum of all the ionic and all axial currents is represented by $I_\Sigma = I_{ION} + I_{axial}$, where I_{axial} stands for the algebraic difference (divergence) of in- and out-going axial currents. In the sequel we will use the notation $u(t) = I_S(t)$ for the stimulation-current waveform. The latter is our *system input*, which will be the leverage to refine in order to achieve desirable outcome - reliable triggering of APs in the excitable system. It is customary in the control literature to denote such a signal $u(t)$.

Thus, all the currents are linked by the first Kirchhoff circuit law:

$$u(t) \equiv I_S(t) = I_C(t) + I_\Sigma[V(t), x(t)] = C_m \dot{V} + I_\Sigma(V, x) \qquad (6)$$

where - in the most general form, I_Σ depends on membrane voltage $V(t)$ and on the state vector of the ionic channels' gate variables. Unless ambiguous, below we will simplify notation by writing $I_\Sigma(V)$.

C_m (typically around 1 $\mu F/cm^2$, [18]) and $V(t)$ (in mV's) are the excitable-membrane's capacitance and potential. Equation (6) can be rewritten as:

$$C_m \dot{V} = u(t) - I_\Sigma(V) \qquad (7)$$

Clearly according to eqn. (7), an outgoing total ionic current *opposes* the effects of cathodic stimulation, since not all of $u(t)$ is employed toward the main goal of maximizing the $V(t)$ growth, which the reader may have also already deduced from the equivalent circuit of Fig. 1. Conversely, ingoing total current *assists* the effects of stimulation. Hence, in such a case $u(t)$ may be *lower*

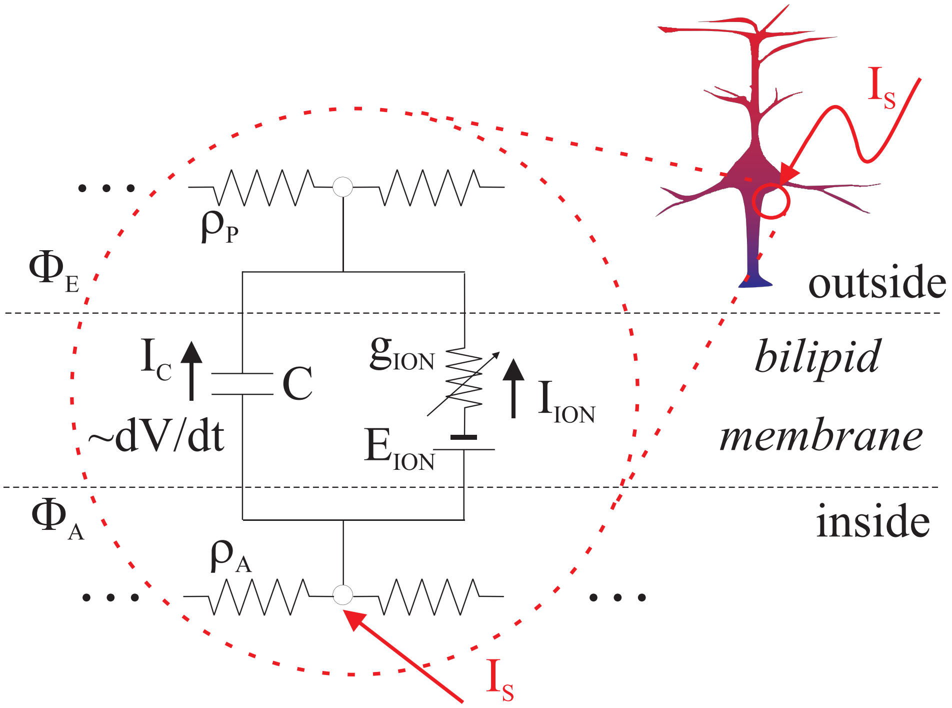

Figure 1. Excitability model *template*: The equivalent circuit represents the simplified electro-dynamics of an excitable membrane. I_S is the intra-cellular stimulation current. $I_C = C\dot{V}$ is the capacitive current. The direction of the latter is for a case of *depolarizing* the membrane's voltage (i.e. the inside of the cell wall becoming more positive). The algebraic sum of all the ionic and all axial currents is represented by $I_\Sigma = I_{ION} + I_{axial}$, where I_{axial} stands for the algebraic difference (divergence) of in- and out-going axial currents.
doi:10.1371/journal.pone.0090480.g001

than when it is estimated assuming the absence of membrane conductivity. Let us elucidate right away by providing typical examples.

Specific Single-compartment (Space-clamp) Models

The models here are *zero-dimensional (0D)*. Their spatial extents are confined to a point. This may be contrasted to the multi-compartment cable-like models that we will discuss later, and whose spatial structure is *one-dimensional (1D)* - i.e. homomorphic to a line.

For single-compartment models there are no axial currents. Hence, $I_\Sigma = I_{ION}$.

Linear Sub-threshold model (LM).

$$I_{ION}(V) = g_m(V(t) - V_r) \tag{8}$$

g_m is the excitable-membrane's resting ($V = V_r \sim -70\ mV$) conductance - in milli-Siemens per unit membrane surface area - e.g. $1\ mS/cm^2$. Substituting $I_{ION}(V)$ from eqn. (8) into eqn. (6) yields a linear first-order model with $\tau = C_m/g_m = R_m C_m$ the familiar expression for the time constant of such a dynamic model. This model predicts a reasonable resting $\tau \approx 1\ ms$.

As pointed out in the introduction, this type of model was extensively used by the ES pioneers, [12]. They were particularly concerned with the derivation of analytic expressions for the experimentally observed *strength-duration* (SD) curves. The latter describe the threshold (minimal) current strength (I_{THR}), which if maintained constant (i.e. through a rectangular waveform) for a given duration T is likely to elicit an AP in excitable-tissue (see the introductory section).

Even if it may account for a significant part of the sub-threshold variation of the membrane's potential, the linear model lacks a paramount feature - it cannot fire AP's as the latter are due to the highly *nonlinear* properties of the excitable-membrane's conductance around and beyond the firing threshold.

The Hodgkin-Huxley-type model (HHM). Hodgkin and Huxley (HH) not only proposed a novel way to model ionic-channels but also introduced ionic-channel-specific parameters to fit experimental data [19]. Since, HH-type models have been proposed for many ionic-channels for cardiac to neuroscience applications.

We present one such model from the literature - [20], which has been used to fit experimental data from the central nervous system and particularly the *neocortex*.

$$I_{ION}(V,x) = g_{Na} m^3 h(V - E_{Na}) + g_K n(V - E_K) + g_{leak}(V - E_{leak}) \tag{9}$$

See Tables 3 and 4, which define all the model's variables and parameter values. We consider specifically the $Na^+_{v1.6}$ sodium channel subtype, to which the axon initial segment (AIS) owes its higher excitability [20,21].

The dynamics of a gate-state variable $x(t)$ (where $x(t)$ stands for one of $m(t)$,$h(t)$,$n(t)$) are described by:

$$\tau_x(V)\dot{x} + x = x_\infty(V) \quad (10)$$

Eqns. (6), (9) and (10) define a system of four coupled ODE's - with respect to the four dynamic variables $[V,m,h,n](t)$.

Further simplification may reduce the model complexity, maintaining only $V(t)$ as the single dynamic variable. Gate-variable states are factored out by introducing appropriate non-dynamic functions of the membrane potential. E.g. in eqn. (9), the fast m gates may be assumed to reach instantaneously $m_\infty(V)$, while the far slower h and n gates remain at their resting values (corresponding to a membrane at its resting equilibrium potential V_r).

The Izhikevich model (IM).

$$I_{ION}(V,w) = w - 0.04V^2 - 5V - 140 \quad (11)$$

This model [22] has a second-order nonlinearity, compared to its predecessor - the BVDP model [23], which contains a cubic nonlinearity. The IM will therefore not auto-limit. As in the BVDP, there is a *slow* second dynamic variable $w(t)$ called the 'recovery current' and its dynamics is described by:

$$\dot{w}/c = bv - w \quad (12)$$

The IM responds to supra-threshold stimulation with a wide variety of AP-firing patterns, depending on the particular choices of parameters. Interested in the sub-threshold regimen, we have chosen the "Spike Latency" set: $b = 0.2, c = 0.02$ [24]. Hence, $\tau_w = 1/c$ is equal to 50 ms. At the time-scale of a single stimulation pulse (lasting at most a few milliseconds), w is virtually a constant.

Here, it may be important to remind the reader that the state of simplest models like the IM needs to be artificially *reset* after an AP event. However in more complex models (e.g. the HHM), channels that are responsible to revert the system to its resting potential will have a significant effect on the optimal waveform. We will see this in more detail in the results section.

Multi-compartment Models

To expand the scope of our analysis and the applicability of its results, it is essential to also address models of AP initiation and propagation along spatial neural structures. A popular example is the McIntyre, Richardson, and Grill model ($MRG'02$). It was originally used to simulate the effects of ES in the peripheral nervous system and specifically the myelinated axons that form nerve bundles [25]. An adapted version of the same model was recently used to simulate the effects of DBS [7].

Myelinated axon has been pinpointed as the most excitable tissue with extracellular stimulation [26–28]. Therefore models like the MRG'02 are of particular interest. Moreover, this model facilitates the illustration of optimality principles as it has only one excitable compartment type - the Ranvier-nodes (RN). The paranodal and other compartments that form the myelinated internodal sections are all modeled as a passive double-cable (due to the myelin sheath that insulates the extracellular periaxonal space) structure, see Fig. 2.

Table 3. Definition and notation for the key HHM variables.

Notation	Variable description and units	Typical value (*1
Potentials, in mV:		
V_m	Membrane voltage	**(*3**
V_{rest}	Membrane resting voltage	-77
E_K	K^+ Nernst potential	-90
E_{Na}	Na^+ Nernst potential	60.0
E_{Leak}	Leak reversal potential	-70
Membrane capacitance, in $\mu F/cm^2$:		
c	Membrane capacitance	1
Maximum **(*2** conductances, in mS/cm^2:		
g_K	K^+ conductance	150
g_{Na}	Na^+ conductance	300
g_{Leak}	Leak conductance	0.033
Currents, in $\mu A/cm^2$:		
I_K	K^+ Ionic Current **(*4**	$g_K \times n \times (V_m - E_K)$
I_{Na}	Na^+ Ionic Current	$g_{Na} \times m^3 h \times (V_m - E_{Na})$
I_{Leak}	Leak Current	$g_{Leak} \times (V_m - E_{Leak})$

Notes:
(*1 Typical values are for the $Na_{v1.6}$ model, [20]; see also Table 4.
(*2 These are dependent on (grow with) temperature, the values listed are for $T = 23^\circ C$.
(*3 Membrane voltage is either at its resting value V_{rest}; is depolarized (grows due to stimulation and/or activated sodium Na^+ ion channels); is repolarized (decays back to V_{rest}, due to the potassium K^+ ion channels).
(*4 Ionic currents depend on both the membrane voltage and the dynamic state of the ion channels' gates. See Table 4.
doi:10.1371/journal.pone.0090480.t003

Table 4. Gate-state dynamics parameters.

Notation	Variable description	Value
Temperature dependence:		
$Q10$	Q_{10} constant **(*1**	2.3
K^+: n-gate **(*2**		
a_n	n-gate max opening rate	0.02
b_n	n-gate min closing rate	0.002
$V_{n,1/2}$	half-min/max in/activation rate voltage	25 mV
k_n	n-gate voltage constant k	9
$Na^+_{v1.6}$: m-gate **(*2**		
a_m	m-gate max opening rate	0.182
b_m	m-gate min closing rate	0.124
$V_{m,1/2}$	half-min/max in/activation rate voltage	41 mV
k_m	m-gate voltage constant k	6
$Na^+_{v1.6}$: h-gate **(*2**		
a_h	h-gate max opening rate	0.024
b_h	h-gate min closing rate	0.0091
$V_{h,1/2,a}$	half-max activation rate voltage	48 mV
$V_{h,1/2,b}$	half-min inactivation rate voltage	73 mV
k_h	h-gate voltage constant k	5
$k_{h,\infty}$ **(*3**	asymptotic gate-state voltage constant k_∞	6.2
$V_{h,1/2,\infty}$	50% open gates voltage	70 mV

Notes:
(*1 Temperature dependence is linear and with a slope $k_T = Q_{10}^{(T-T_0)/10}$, where $T_0 = 23^\circ C$.
(*2 For a given gate type y of the K^+ and $Na^+_{v1.6}$ ionic channels, the fractions of open and closed gates are given by the general (Boltzmann-Energy like) *template* formulae:

$$\alpha_y(w) = a_y w/(1-\mathbf{e}^{-w/k_y}) \quad \beta_y(w) = -b_y w/(1-\mathbf{e}^{w/k_y}) \text{ where}$$

$w = V_m - V_{1/2}$.
Thus, the corresponding *rates* of opening $d\alpha_y/dw$ and closing $d\beta_y/dw$ are sigmoidal functions of w s.t.

$$\lim_{w\to-\infty} \alpha_y = \lim_{w\to\infty} \beta_y = 0 \quad \lim_{w\to\infty} \alpha_y = a_y \quad \lim_{w\to-\infty} \beta_y = -b_y$$

The actual position of the inflection point ($w=0$) is determined by the $V_{1/2}$ parameter. For the m and n gates, by the l'Hospital-Bernoulli rule, it can be seen that at $V_m = V_{1/2}$, the opening or closing rates attain half of their max or min, respectively.
(*3 For the inactivating gate h of the $Na^+_{v1.6}$ ionic channel:

$$h_\infty(V) = 1/(1+\mathbf{e}^{w_h/k_{h,\infty}}) \quad w_h = V_m - V_{h,1/2,\infty}$$

doi:10.1371/journal.pone.0090480.t004

The RN compartment is a model of the HH-type:

$$I_{ion}(V,x) = g_{Na,f} m^3 h(V-E_{Na}) + g_{Na,p} p^3 (V-E_{Na}) + g_K n^4 (V-E_K) + g_{leak}(V-E_{leak}) \tag{13}$$

Here two different Na^+ ion channel subtypes are modeled (please see Table 5 for all the details). The *fast* subtype (with maximum conductance parameter $g_{Na,f}$) is controlled by the opening m and closing h gate states. The *persistent* subtype (with maximum conductance $g_{Na,p}$) is controlled by the p gates. As its name suggests, it has *no* gate-inactivating states and is *non-inactivating*. In addition, this model has *very* slow s gates, associated to its K^+ ion channel and *very* fast m gates.

Below we call a *fixed point* (FP) every V_{FP} value s.t. $I_{ION}(V_{FP}) = 0$. From eqn. (7) with $u = 0$,

$$\dot{V}\big|_{V_{FP}} = 0$$

The nonlinear dynamics behavior of the RN compartment taken in isolation is quite unlike that of the specific single-compartment HHM example we provided above. None of its four FPs are stable. Around its *unstable* 'resting' state ($V_r = -80\ mV$), the zero-dimensional RN's of MRG'02 model yield *depolarizing*

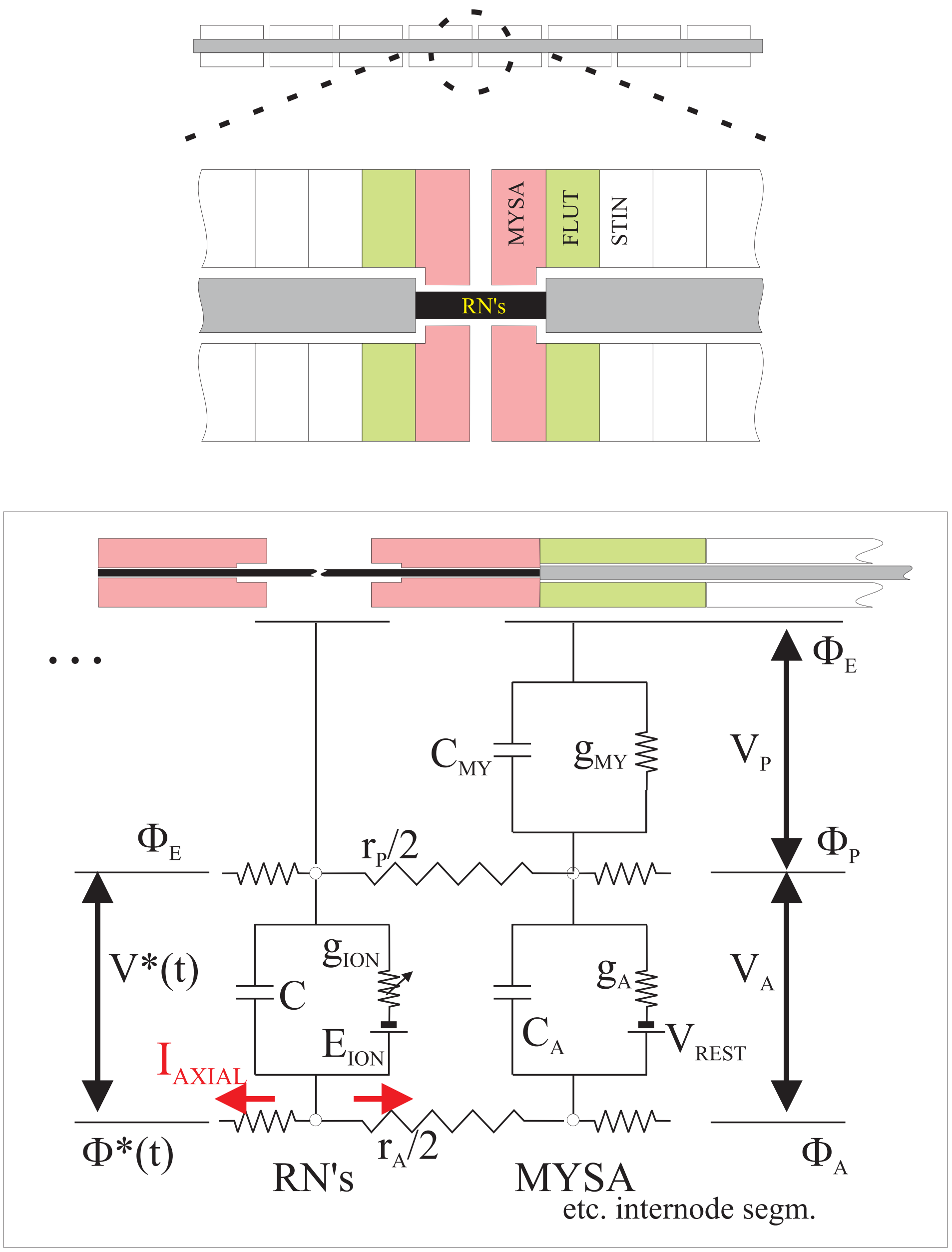

Figure 2. The MRG'02 myelinated axon model (See also Table 4) Box: Equivalent circuit for current injection into the center RN (#1).
doi:10.1371/journal.pone.0090480.g002

ionic current. I.e. not only does I_{ION} not resist moving away from the resting state, but it actually contributes to automatic firing, with or without any external current!

The addition of the passive myelinated spatial structures around the RN's makes the resting state stable, and the problem at hand (of identifying the LAP-optimal ES waveforms) tractable only within a spatial structure. However, this also comes with bonuses. First, the active-passive association brings a very clear-cut picture of the factors at hand that influence AP initiation and propagation. Second, the myelinated double-cable has a very low spatial constant, which provides for a straightforward extension of the single-compartment analysis.

Namely, consider the second term in the more general expression for $I_{\Sigma} = I_{ION} + I_{axial}$ in eqn. (7). Since around the resting state I_{ION} is always there as a depolarizing factor, it is I_{axial} that needs to be closely considered, see Box in Fig. 2.

The numerical results presented for the MRG'02 in the literature [7,8] often target the mid-cable (center) RN in their ES simulations. This motivated us to use of the method of mirrors to double the model's dimensions at the same computational cost. We consider a long axon (with 41 RN's), which has a relatively low length constant ($\lambda^2 \sim 1/(g_a \rho_a)$). See also Tables 4 and 5. For the RN's $\lambda = 167.5\ \mu m$ vs respectively 2129.7 and 443.2 μm, for the myelinated and the MYSA (paranode) sections. These are paired to significant differences in the passive membrane time

Table 5. MRG'02 double-cable model-axon electrical parameters.

Notation	Parameter description	Value
Shared parameters:		
V_{rest}	Resting potential	$-80\ mV$
ρ_a	Axoplasmic resistivity	70 Ω
ρ_p	Periaxonal resistivity	70 Ω
Nodal compartments:		
c_n	Membrane capacitance	2 $\mu F/cm^2$
E_K	K^+ Nernst potential	$-90\ mV$
E_{Na}	Na^+ Nernst potential	50.0 mV
E_{Leak}	Leak reversal potential	$-90\ mV$
$g_{K,s}$	Maximum slow K^+ conductance with opening s and *no* closing gate states	0.08 S/cm^2
$g_{Na,f}$	Maximum fast Na^+ conductance with opening m and closing h gate states	3.0 S/cm^2
$g_{Na,p}$	Maximum persistent Na^+ conductance with opening p and *no* closing gate states	0.01 S/cm^2
g_{Leak}	Leak conductance	0.007 S/cm^2
Internodal compartments:		
c_i	Membrane capacitance	2 $\mu F/cm^2$
E_{Psv}	Passive-compartment Nernst potential	
Passive (leak) membrane conductance by segment type:		
g_a	MYSA	0.001 S/cm^2
g_f	FLUT	0.0001 S/cm^2
g_i	STIN	0.0001 S/cm^2
Myelin parameters:		
c_{my}	Capacitance	0.1 $\mu F/cm^2$
g_{my}	Conductance	0.001 S/cm^2

Notes:
See also Table 6.
doi:10.1371/journal.pone.0090480.t005

constant ($\tau \sim c_i/g_a$). For the RN's $\tau = 0.29\ ms$ vs respectively 20 and 2 ms, for the myelinated and paranode sections. The cable end-conditions are formed by virtual compartments with membrane at rest $V_r = -80\ mV$. This choice is further motivated by the results of model simulations - namely the relatively little spread of potentials at the end of stimulation lasting up to a few milliseconds (see Fig. 3).

We studied extensively all the published accounts of the MRG'02 model and its use for ES modeling [7,8,25]. We also carefully compared parameter values (see Tables 5 and 6) to the ones in the official NEURON models database (senselab.med.yale.edu/modeldb/ShowModel.asp?model=3810).

Our model implementation originally for [29,30] was done in Matlab (the Mathworks, ver. 7 and above). The code uses CVODES (the Lawrence Livermore National Laboratory, Release 2.7.0) to reliably and robustly solve the related multi-dimensional system of ODEs. The implementation was validated through extensive comparisons and personal correspondence with the authors of the original model - W.M. Grill [31] and A.G. Richardson, regarding specifically the mismatch between the 2002 publication and its NEURON implementation.

Preliminary Analysis: On the Existence of the AP-firing Threshold

The above ionic-current descriptions differ largely in form and complexity. Yet each of them is capable of capturing some of the essential dynamics properties of excitable living tissues.

In order to elicit an AP through electric stimulation, the membrane's potential $V(t)$ needs to first be driven (*depolarized*, $\dot{V} > 0$) to some threshold value V_{THR}, beyond which *assisting* ionic channels are massively engaged to produce the AP upstroke without the need of any further ES intervention. From eqn. (6) in order to do so, the stimulation waveform needs to be positive and superior to $I_\Sigma(V,x)$ at most times - i.e. $u(t)$ needs to overcome the *opposing* currents.

A V_{THR} value is hiding inside each of the above nonlinear flavors of $I_\Sigma(V,x)$. Predictably, it is easiest to find the V_{THR} value associated with the IM. Above we saw that the variable w in the IM reacts *slowly* to changes in V. Hence, one may approximate it by its value at rest: $w_r = bV_r$. The *resting* membrane potential V_r is then obtained from the condition $I_{ion,0}(V_r) = 0$, where the subscript 0 indicates that we have assumed $w(t) = w_r$.

The *resting* potential V_r is one of the zeroes of the 2nd-order polynomial in $V(t)$, which characterizes the ionic current. The second zero is V_{THR}. Beyond this threshold the total ionic current

switches its sign. So eqn. (11) becomes:

$$\begin{aligned} I_{ION,0}(V) &= -0.04V^2 - 5V + bV_r - 140 \\ &= -0.04(V - V_r)(V - V_{THR}) \end{aligned} \tag{14}$$

Hence, $V_r = -70$ mV and the *resting* threshold is $V_{THR,0} = -55$ mV.

We will utilize this simple nonlinear model to complete the picture. If $w(V,t) > w_r$ - i.e. the membrane is *not* at rest, the point where the total ionic current $I_{ION}(V)$ switches sign is shifted rightward toward a *higher* V_{THR} value. For example, for very long durations $T \rightarrow \infty$, $w \rightarrow bV$:

$$\begin{aligned} I_{ion,\infty}(V) &= -0.04V^2 + (b-5)V - 140 \\ &= -0.04(V - V_r)(V - V_{THR}) \end{aligned} \tag{15}$$

The subscript ∞ indicates that we have assumed $w(t) = bV(t)$. Predictably, this does not affect the resting potential, since $I_{ion,\infty}(V_r) = I_{ion,0}(V_r)$. However, $V_{THR,\infty} = -50$ mV is higher than the resting threshold $V_{THR,0}$.

This reflects the lowering of excitability shortly after an AP, and once the post-AP membrane re-polarization takes place. This is known as *refractoriness*, which can be either *absolute* - i.e. no AP can be elicited *regardless* of how large the stimulation, or *relative* - i.e. larger stimulation current is required - to reach a higher threshold V_{THR}.

Some models of the HH-type have even more complex $I_{ION,\infty}(V)$ and thence V_{THR} behavior. This complexity is due to the multiple gate states, which may have very different *time constants* and hence reach their asymptotic states at different times. In addition, the HH models involve *inactivating* sodium (Na^+) channels. Hence, excitability may be conditional on attaining the firing threshold within a *specific* time window. Then V_{THR} may exist only with durations $\ll \infty$. Hence, even over arbitrarily long duration, an arbitrarily low (non-zero) current may *never* elicit AP's, and may also damage the tissues and the electrodes as irreversible chemical reactions take place.

So, *wide* stimulation pulses lasting well over some critical duration T_{CR} may not be able to elicit any AP. This is due to the comparable temporal scales of duration T_{STIM} and the time constant τ_{ion} of the closing gates associated with depolarizing ionic currents and of the opening gates associated with re-polarizing currents.

Therefore, let us assume that the excitable-membrane's potential is at its resting value V_r. Hence, *in principle* an action potential (AP) can be elicited by stimulation of the *fixed* duration $T < T_{CR}$. Therefore stimulation takes place over a finite time-horizon.

Finite-Horizon Optimal-Control (FHOC)

In this approach, the current waveform is the unknown system input signal complying with specific optimality criteria. The optimal pattern $u^*(t)$ for $t \in [0,T]$ is sought as a solution of the following constrained minimization problem:

$$u^* = \arg\min \left\{ \Theta(\mathbf{X}(T)) + \int_{t=0}^{T} f_0(\mathbf{X},u)dt \right\} \tag{16}$$

$$\frac{d}{dt}\mathbf{X} = \mathbf{F}(\mathbf{X},u) \qquad \forall u(t) \in [L,R]$$

where L and R are the constant lower and upper bounds on the values for each $u(t)$ sought.

The computational model's dynamical system is introduced in the optimization problem of eqn. (16) in the form of a set of equality constraints. The *vector* function $\mathbf{F}(x,u) \in \mathbf{R}^n$ describes the dynamics of the *array* of system state-variable trajectories $x_i(t), i = 1 \ldots n$, resulting from given initial state $\mathbf{X}(0)$ and control signal u.

The example developed in the Results section uses the Izhikevich model - eqns. (6) and (11) - with $n = 2$.

The minimized functional, contains the integration term $f_0(\mathbf{X},u)$ and a final-time (also known as penalty) term $\Theta(\mathbf{X}(T))$ - pulling toward the desired final state $\mathbf{X}^*(T)$. The specific f_0 expression yields minimum electric stimulation power:

$$f_0(\mathbf{X},u) = u(t)^2/2 \tag{17}$$

The penalty term is a convenient way to express the desirable stimulation's outcome - the membrane voltage reaching some pre-defined threshold-level V_{THR}:

$$\Theta(\mathbf{x}(T)) = \frac{K_{penalty}}{2(V_{THR} - V(T))^2} \tag{18}$$

Using a general constrained parametric optimal-control approach (e.g. [32]), the objective and equality constraints in eqn. (16) are combined into the *Lagrangian*:

$$\begin{aligned} \mathcal{L} &= \Theta(\mathbf{x}(T)) + \int_{t=0}^{T} \left[f_0(\mathbf{X},u)dt - \lambda'(\frac{d}{dt}\mathbf{X} - \mathbf{F}(\mathbf{X},u)) \right] dt \\ &= \Theta(\mathbf{x}(T)) + [\lambda'\mathbf{X}]_{t=0}^{T} + \int_{t=0}^{T} \left[H + \frac{d}{dt}\lambda'\mathbf{X} \right] dt \end{aligned} \tag{19}$$

where $\lambda(t)$ are the *Lagrange multipliers*, associated to *each* of the n equality constraints in eqn. (16) and $(\cdot)'$ stands for the vector-matrix *transpose* operator. $H = f_0(x,u) + \lambda'\mathbf{F}(\mathbf{X},u)$ is known as the *Hamiltonian*.

The *necessary conditions* for optimality require that all partial derivatives of the Lagrangian by the system states vanish at the optimal solution to the problem of eqn. (16) - i.e.:

$$\frac{\partial \mathcal{L}}{\partial \mathbf{X}(t)} = 0 \qquad \forall t \in [0,T] \tag{20}$$

Here the 'vector-matrix' notations $\partial\phi/\partial\mathbf{X}$ or $\partial\mathbf{F}/\partial\mathbf{X}$, where $\mathbf{X} \in \mathbf{R}^n$, mean respectively $\partial\phi/\partial x_i$ or $\partial f_i/\partial x_j$, $\forall i,j = 1 \ldots n$.

This development is known as mathematical *sensitivity analysis* and its main purpose is to reveal the impact of a given system parameter (such as $u(t)$ or its initial state $\mathbf{X}(0)$) on the resulting dynamics.

From eqns. (19) and (20):

$$\frac{d}{dt}\lambda = -\frac{\partial H}{\partial \mathbf{X}} \tag{21}$$

$$\lambda(T) = \frac{\partial \Theta}{\partial \mathbf{X}(T)}$$

where

$$\frac{\partial H}{\partial \mathbf{X}} = \frac{\partial f_0}{\partial \mathbf{X}} + \frac{\partial \mathbf{F}'}{\partial \mathbf{X}}\lambda$$

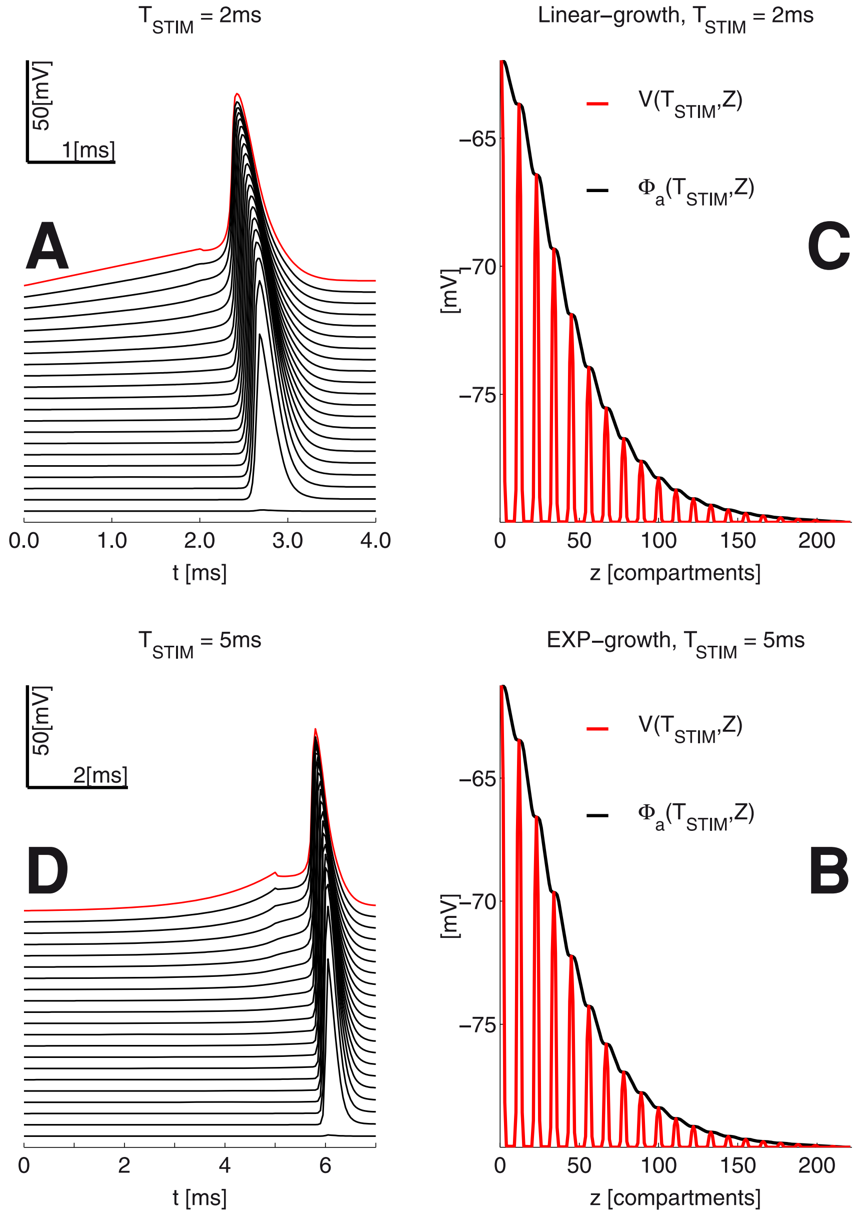

Figure 3. Propagating AP's and spatial profile of the membrane voltage $V(t,z)$ & intracellular potential $\Phi_a(T_{STIM},z)$ (at the end of stimulation, please also see Fig. 2); z is the 1D axonal spatial coordinate. The peaks of V at the Ranvier nodes are due to the direct exposure to the extracellular medium, which is unlike that of the myelinated sections in the double-cable MRG'02 model.
doi:10.1371/journal.pone.0090480.g003

Notice that eqn. (21) describes the *adjoint* dynamic system iterated in *reverse* time with a *terminal* condition provided by the derivative of the $\theta(\mathbf{X}(T))$ term. To solve the ODE system of eqn. (21), the achieved forward dynamics of eqn. (16) needs to be already computed.

Similarly, all partial derivatives of the Lagrangian by $u(t), \forall t \in [0,T]$ vanish at the optimal solution to the problem of eqn. (16) - i.e. $\forall k = 0 \dots m-1$:

$$\frac{\partial \mathcal{L}}{\partial u_k} = \int_{t=k\Delta t}^{T} \frac{\partial H}{\partial u} dt \qquad (22)$$

where Δt is the sampling time, $u_k = u(k\Delta t)$ and

$$\frac{\partial H}{\partial u} = \frac{\partial f_0}{\partial u} + \frac{\partial \mathbf{F}'}{\partial u} \lambda$$

Hence, eqn. (22) yields all components of the *gradient* w.r.t. $u(k\Delta t)$, which enables the use of gradient-based quasi-Newton search routines (e.g. fmincon from the Matlab optimization toolbox).

Moreover, one sees from eqn. (19) that the array $\lambda(0)$ is the sensitivity (i.e. the gradient) w.r.t. initial state $\mathbf{X}(0)$, i.e.:

Table 6. MRG'02 double-cable model-axon geometric parameters, in μm.

Notation	Parameter description	Value
Shared parameters:		
D	Fiber Diameter	16.0
ΔZ	Node-node separation	1500
N_{my}	Number of myelin lamellae	150
Nodal compartments:		
L_n	Node length	1.0
d_n	Node diameter	5.5
MYSA (myelin attachment paranode)		
L_M	length	3.0
d_M	diameter	5.5
δ_M	periaxonal width (Membrane-to-Myelin gap)	0.004
FLUT compartments (main section of paranode)		
L_F	length	60.0
d_F	diameter	12.7
δ_F	periaxonal width	0.004
STIN compartments (internodal section, 3+3 total in 1 internode, see Fig. 2)		
L_S	length	228.8 **(*1**
d_S	diameter	12.7
δ_S	periaxonal width	0.004

Notes:
(*1 $L_S = \dfrac{\Delta Z - L_n - 2(L_M + L_F)}{6}$.

doi:10.1371/journal.pone.0090480.t006

$$\lambda(0) = \frac{\partial \mathcal{L}}{\partial \mathbf{X}(0)}$$

A boundary-value problem (BVP), with known *initial* conditions for $\mathbf{X}(0)$ and *terminal* conditions for $\lambda(T)$, is solved numerically. However, it should also be noted that such solutions may also converge to shallow *local* minima. For example, the Newton search is guaranteed to produce the 'true' solution when the problem at hand involves a quadratic cost. Here the objective function not only may be non-quadratic, but also may be non-convex in some manifolds of its high-dimensional parametric space.

Above we described the *continuous-time* FHOC. The CVODES toolbox readily provides adjoint sensitivity analysis (ASA) capabilities. FHOC is one of the common applications of the latter. Analogously, a *discrete-time* version may be formulated and solved (see the Results section, where a specific example is developed).

Solving the Problem Analytically: The PLA in ES

Through calculus of variations, here we establish a general form for the energy-optimal current waveform $u^*(t)$. This approach applies the Principle of Least Action to ES.

Let us assume that $T \ll \tau_{ION}$, where τ_{ION} is the time-constant that determines the behavior of the *slow* gate states of the modeled ionic-channels. Hence, the *fast* gate states may be approximated by their asymptotic values $x_\infty(V) = \lim_{t\to\infty} x(t|V)$, while the *slow* gate states - by their resting values $x_0 = x_\infty(V_r)$.

Then an AP can readily be evoked by stimulation from the resting state, and the threshold potential V_{THR} to reach at time T is finite and assumed (without loss of generality) to be known. The energy-efficiency of driving the excitable-tissue membrane potential $V(t)$ from its resting value V_r to V_{THR} through a stimulation of *fixed* duration T satisfies:

$$u^*(t) = \arg\min_u P(u) \qquad P(u) = 1/2 \int_0^T [u(t)]^2 dt \tag{23}$$

Since from eqn. (6), $u(t) = C_m \dot{V} + I_\Sigma(V)$:

$$P(u) = S(V|T,u) = 1/2 \int_0^T \left[C_m \dot{V}(t) + I_\Sigma(V) \right]^2 dt \tag{24}$$

As done in the calculus of variations let us perturb the energy-optimal time-course $V^*(t)$ by the infinitesimal perturbation $\epsilon\eta(t)$, where $\eta(t)$ is an arbitrary function of time and ϵ is an infinitesimal scalar.

$$\begin{aligned} V(t) &= V^*(t) + \epsilon\eta(t) \\ I_\Sigma(V) &= I_\Sigma(V^*) + \epsilon \frac{\partial I_\Sigma(V^*)}{\partial V} \eta(t) \end{aligned} \tag{25}$$

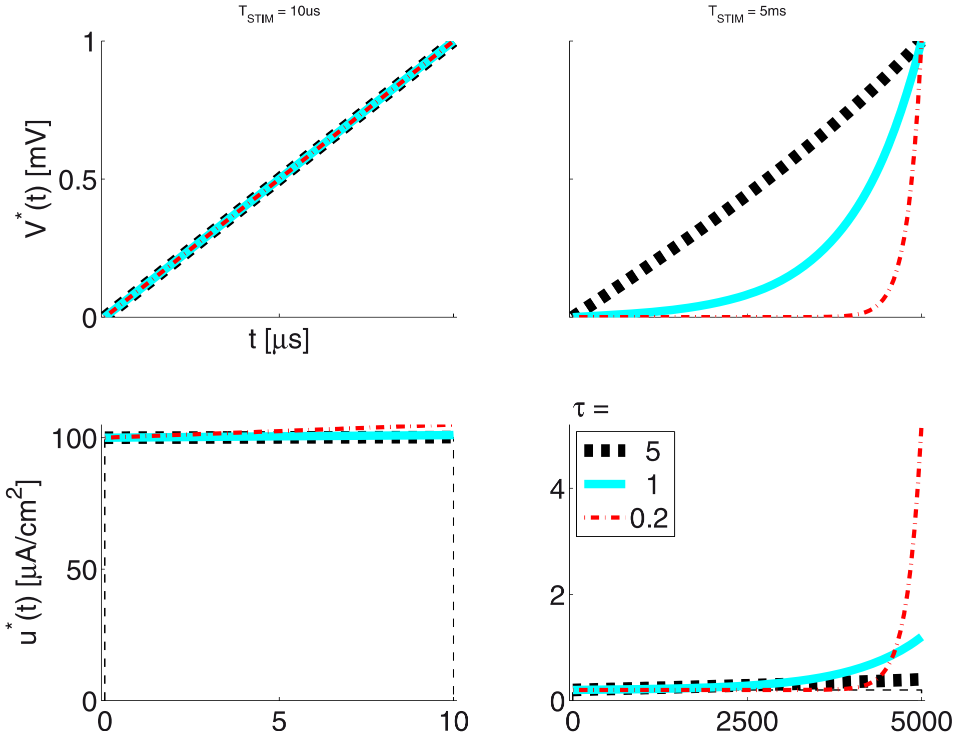

Figure 4. LAP energy-optimal $V^*(t)$ and $u^*(t)$ for the LM: for T_{STIM} respectively 10 μs and 5 ms; the time constant $\tau = C/g$ was varied as indicated in the legend; membrane capacity was constant - $C = 1\ \mu F/cm^2$, while membrane (leak) conductance g was respectively 0.2, 1 and 5 mS/cm^2; The 3 solutions shown correspond to the nominal $\tau = 1\ ms$ (cyan trace) or 5-fold shorter (thin red dash-dot), or 5-fold longer (thick dashed black) τ respectively; (thin dashed black) rectangular pulse with amplitude $k^* = (V_{THR} - V_r)/T_{STIM}$.
doi:10.1371/journal.pone.0090480.g004

From eqn. (25), $\forall t \in [0, T]$ the integrand in eqn. (24) becomes:

$$\begin{aligned}(C_m\dot{V} + I_\Sigma(V))^2 &= (C_m\dot{V}^* + I_\Sigma(V^*))^2 \\ &+ 2\epsilon(C_m\dot{V}^* + I_\Sigma(V^*))(C_m\dot{\eta} + \eta I'_\Sigma(V^*)) \\ &+ \epsilon^2(C_m\dot{\eta} + \eta I'_\Sigma(V^*))^2\end{aligned} \tag{26}$$

From eqns. (24) and (26), and since $u^*(t) = C_m\dot{V}^* + I_\Sigma(V^*)$.

$$\begin{aligned}P(\epsilon) &= S(V^*) + \epsilon\int_0^T u^*(t)(C_m\dot{\eta} + \eta I'_\Sigma(V^*))dt \\ &+ \epsilon^2 F(V^*, \eta)\end{aligned} \tag{27}$$

The necessary condition for $S(V)$ to have a *minimum* at $\epsilon = 0$ for *any* $\eta(t)$ is:

$$G_\epsilon = P'(\epsilon)|_{\epsilon=0} = \int_0^T u^*(t)(C_m\dot{\eta} + \eta I'_\Sigma(V^*))dt = 0 \tag{28}$$

To deal with the $u^*(t)\dot{\eta}$ term of eqn. (28), it is integrated by parts :

$$G_\epsilon = C_m[u^*(t)\eta(t)]_0^T - \int_0^T [C_m\dot{u}^* - u^* I'_\Sigma(V^*)]\eta(t)dt = 0 \tag{29}$$

Since the perturbation $\eta(t)$ respects the boundary-value problem (BVP) with known *initial* and *terminal* conditions for $V^*(t)$ - i.e. $\eta(0) = \eta(T) = 0$, then the first RHS term above vanishes. Hence, the only way that eqn. (29) will hold for *any* $\eta(t)$ is that we have the Euler-Lagrange-type equation:

$$C_m\dot{u}^* = I'_\Sigma(V^*)u^* \tag{30}$$

Equation (30) can also be attained directly using the continuous version of the standard OC formalism [32] (please see also the just presented FHOC subsection above).

Here the *Hamiltonian* is.

$$H = u^2/2 + \lambda(u - I_\Sigma(V))/C_m. \tag{31}$$

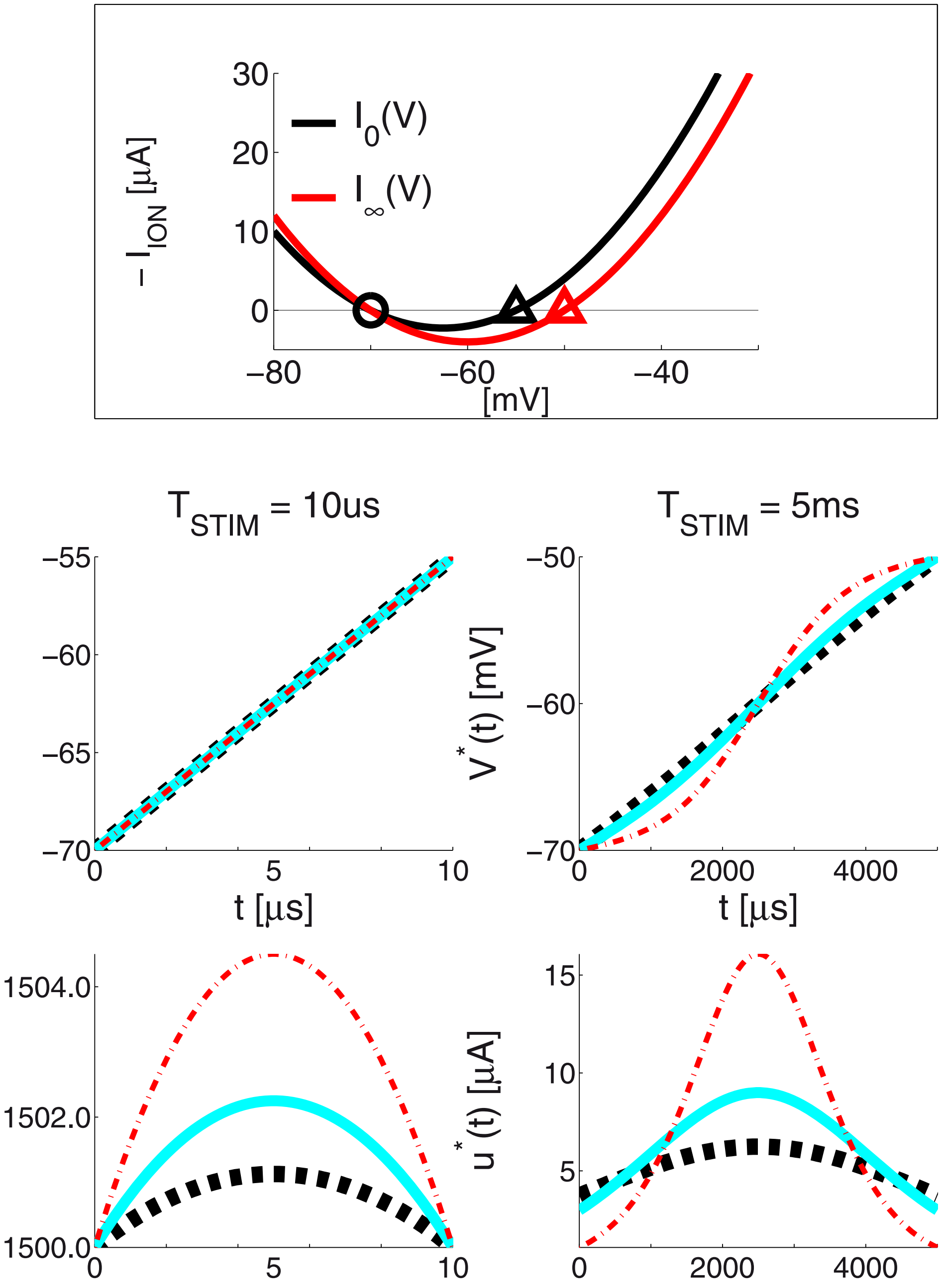

Figure 5. LAP optimal waveforms $V^*(t)$ and $u^*(t)$ for the 0D IM: The 3 solutions shown correspond to the nominal IM opposing current (cyan trace), twice higher (thin red dash-dot), or twice lower (thick dashed black) I_Σ respectively. The $I_{ION,0}(V)$ approximation of the ionic current is used for a case of very short duration ($T_{STIM}=10\ \mu s$) and the $I_{ION,\infty}(V)$ approximation is used for a case of long duration ($T_{STIM}=5\ ms$). It is important to notice that - as with the LM model above, $u^*(t)\approx k^*+I_{ION}(V)$, where $k^*=(V_{THR}-V_r)/T$ (see the Box) **Box:** Resting-state $I_{ION,0}(V)$ and asymptotic-state $I_{ION,\infty}(V)$ ionic currents for the 0D IM; Markers are inserted at the resting and threshold membrane-voltage points, respectively $V_{REST}=-70$, $V_{THR,0}=-55$ and $V_{THR,\infty}=-50\ mV$.
doi:10.1371/journal.pone.0090480.g005

The *necessary conditions* for optimality require that.

$$\partial H/\partial u = 0 \qquad (32)$$

$$\dot{\lambda} = -\partial H/\partial V \qquad (33)$$

From eqns. (32) and (31) $\lambda/C_m=-u$. Then from eqn. (33).

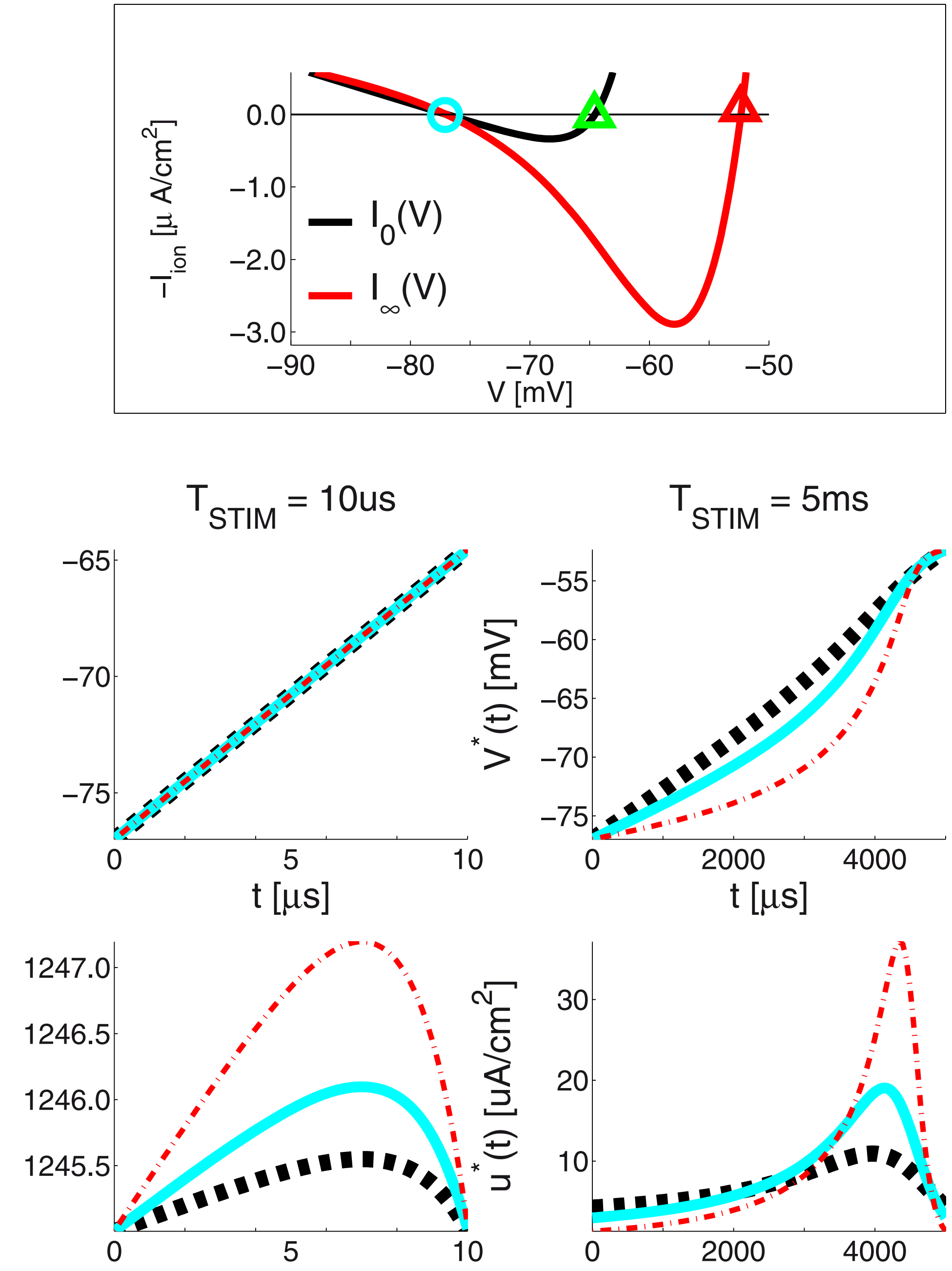

Figure 6. LAP optimal waveforms $V^*(t)$ and $u^*(t)$ for the 0D HHM: The $I_{ION,0}(V)$ approximation of the ionic current is used for a case of very short duration (T_{STIM} = 10 μs) and the $I_{ION,\infty}(V)$ approximation is used for a case of long duration (T_{STIM} = 5 ms) (see the Box)**.** As with the IM, bvp4c was used to numerically solve the BVP of eqn. (34). The figure follows a quite similar format to Fig. 5. $I_\Sigma(V)$ can also be assumed higher or lower. All the maximal ionic conductances in the HHM (see also Table 3) are temperature-dependent and are linearly proportional to the coefficient k_T. The 3 solutions shown correspond to the ionic current at $T^oC[37^oC]$ (cyan trace), twice higher (thin red dash-dot), or twice lower (thick dashed black) I_Σ respectively. From eqn. (42) we can see that $k_T = 1.6047$ (half the nominal) at $28.7^\circ C$, and $k_T = 6.4188$ (twice the nominal) for at $45.3^\circ C$. **Box:** Resting-state $I_{ION,0}(V)$ and asymptotic-state $I_{ION,\infty}(V)$ ionic currents for the 0D HHM; Markers are inserted at the resting and threshold membrane-voltage points, respectively $V_{REST} = -77\ mV$, $V_{THR,0} = -64.55\ mV$ and $V_{THR,\infty} = -52.35\ mV$.
doi:10.1371/journal.pone.0090480.g006

$$C_m\dot{u} = \partial H/\partial V = -\lambda/C_m I'_\Sigma(V) = uI'_\Sigma(V)$$

which is the same as eqn. (30).

From eqns. (6) and (30) we have that.

$$\dot{u}^*(t) = C_m\ddot{V}^* + I'_\Sigma(V^*)\dot{V}^* = I'_\Sigma(V^*)u^*/C_m$$

and thence:

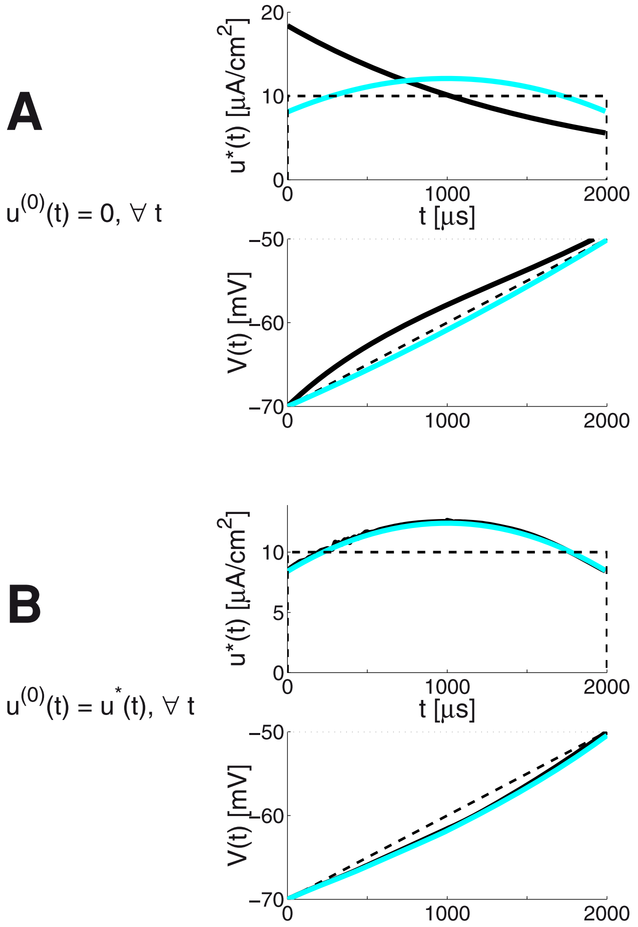

Figure 7. The LAP vs or with numerical optimisation for the 0D IM, with $T_{STIM}=2$ ms: see also Fig. 5 which shows that an initial guess $\hat{u}^{*}(t)$, based on the *linear*-growth rate $k^{*}=(V_{THR}-V_r)/T_{STIM}$ is still valid with $T_{STIM}=2$ ms and $V_{THR}=-50$ mV. panel A: *discrete-time* IM and FHOC **panel B:** *continuous-time* IM and FHOC, using CVODES adjoint sensitivity analysis capabilities **upper plots:** (dashed black) a rectangular pulse with amplitude k^{*}; (thick cyan) the LAP $\hat{u}^{*}(t)=k^{*}+I_{ION,\infty}(V)$; (thick black) the best FHOC $u(t)$ **lower plots:** (dashed black) *linear*-growth evolution of the membrane potential from V_r at $t=0$ to V_{THR} at $t=T_{STIM}$; (dotted gray) the desired threshold value $V_{THR}=-50$ mV; (thick cyan) the resulting LAP $V^{*}(t)$; (thick black) the resulting FHOC $V(t)$.
doi:10.1371/journal.pone.0090480.g007

$$C_m^2\ddot{V}^{*}=I'_{\Sigma}(V^{*})[u^{*}-C_m\dot{V}^{*}].$$

And finally, from eqn. (6).

$$C_m^2\ddot{V}^{*}=I_{\Sigma}(V^{*})\times\frac{\partial I_{\Sigma}(V^{*})}{\partial V} \tag{34}$$

Equation (34) is a rather simple system of ordinary differential equations (ODE) that can *readily* be solved for a given current model $I_{\Sigma}(V^{*})$ to compute the energy-optimal membrane voltage profile $V^{*}(t)$. The energy-efficient current waveform $u^{*}(t)$ is then computed from eqn. (6).

In the Results section below we illustrate the use of eqn. (34) with several frequently encountered current models.

Results

Here, we first derive some key analytical results using the simplest and clearest models. We then identify generally applicable optimality principles. Finally, we demonstrate how these principles apply also to more complex and realistic models and their simulations.

Part I - Specific Point-model Results, Applying the LAP

For the zero-dimensional (single-compartment, space clamp) models introduced in the Methods, here we describe the LAP-optimal waveforms $V^{*}(t)$ and $u^{*}(t)$, stemming from the general (model-independent) LAP result of eqn. (34).

These simple cases readily illustrate some rather key optimality principles resulting from a LAP perspective. We will discuss these optimality principles as we go, and will summarize them at the end of this subsection.

Linear sub-threshold model. Replacing $I_{\Sigma}(V^{*})$ in eqn. (34) with $I_{ION}(V)$ from eqn. (8):

$$\tau^2\ddot{V}=V \tag{35}$$

$\tau=C_m/g_m=R_mC_m$ is the membrane's time constant and for expediency $V\equiv V^{*}$ and $V_r=0$.

The general solution of eqn. (35) is:

$$V(t)=C_1\mathbf{e}^{\frac{-t}{\tau}}+C_2\mathbf{e}^{\frac{t}{\tau}} \tag{36}$$

Given the boundary conditions $V(0)=0$ and $V(T)=V_{THR}$:

$$V^{*}(t)=V_{THR}\frac{\sinh(t/\tau)}{\sinh(T/\tau)} \tag{37}$$

A result similar to eqn. (37) is obtained by [33], using a slightly different (less direct or general) optimal-control approach.

From eqn. (37) one can see that $V^{*}(t)/V_{THR}=\sinh(t/\tau)/\sinh(T/\tau)\sim t/T$ - i.e. it has a *linear* rise, especially with $T\ll\tau$. Here $T=100$ μs and $\tau=1$ ms (computed using *typical* values from the literature for $g_m=1$ mS/cm^2 and $C_m=1$ $\mu F/cm^2$).

Figure 4 presents the LAP energy-optimal stimulation profiles $V^{*}(t)$ and $u^{*}(t)$ for a short and a long stimulus duration T_{STIM} and three membrane time constant τ values.

Before we go on, it is useful to investigate the conditions for a growing exponent (GE) waveform to outperform the SQR waveform.

First, $u^{*}_{GE}(t)$ has a very rapid rise. Hence, its optimal duration T^{*}_{GE} will be short. Second, it is noteworthy that in [33] $\tau=30.4$ micro-seconds! Hence, injected current rapidly leaks out. However even with the above extreme τ value, at its optimal duration T^{*}_{SQR} the SQR wave does just 22% worse, which means that the SQR is among the best candidates for its robustly good performance.

Second, in multiple cases, the energy-optimal LAP waveform $u^{*}(t)$ looks a lot like a 'classical' rectangular waveform. From eqn. (8), we may also see that, with $V_r=0$, $V_{THR}=1$, the max. value of $I_{ION}(V)$ is equal to 1 and is attained as the membrane potential reaches the threshold $V(T)=V_{THR}$. If we then replace

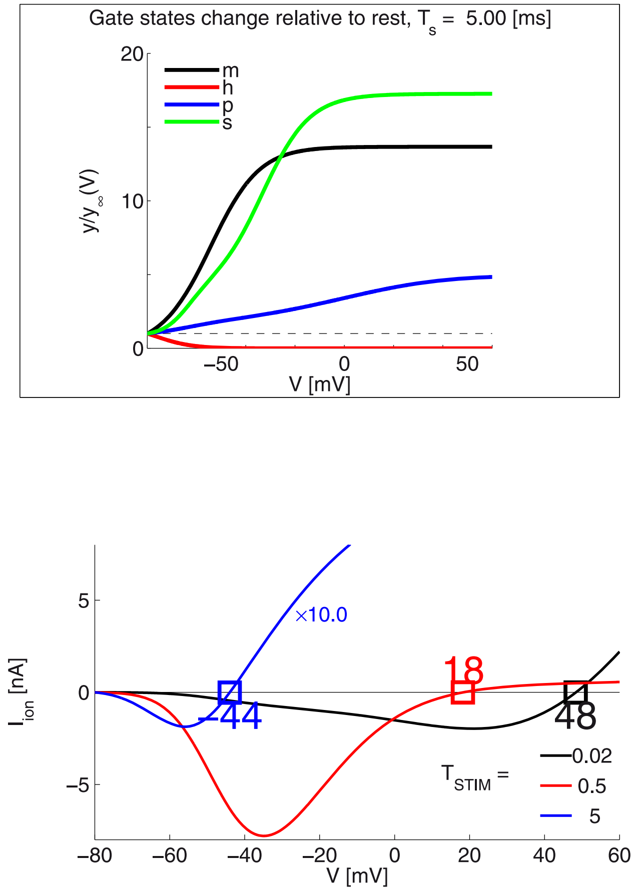

Figure 8. The MRG'02 model: Toward upper bounds on $V_{THR}(T_{STIM})$: the figure presents a family of ionic current $I_{ION}(V,Z)$ approximations at the target site ($Z=0$), for a set of durations T_{STIM}. For each of the durations it is assumed that the membrane voltage trajectory $V(t)$ evolves according to a linear ramp from rest V_r to threshold V_{THR} (the unknown). For each V value on the horizontal (independent-variable) axis of the figure, a $V(t)=kt$ ramp was assumed and the corresponding ionic current $I_{ION}(V)$ was computed, based on approximate gate states (see the Box). Note: for the sake of better visibility, a $\times 10$ gain is applied to the approx. $I_{ION}(V)$ for the case of $T_{STIM}=5$ *ms*. **Box:** For a chosen $T_{STIM}=5$ *ms* and as $V(t)$ is linearly ramped up, for each gate state the plots show the ratio $\hat{x}(V,T)/x_\infty(V)$, where $\hat{x}$ is given by eqn. (46) to its asymptotic value - both functions of V. *Legend for gate states:* opening m and closing h gates for the *fast* Na^+ ion-channel subtype; p *persistent* Na^+ channel gates; s *slow* K^+ gates.
doi:10.1371/journal.pone.0090480.g008

$I_{ION}(V)\approx 0$ in eqn. (6), we see that a waveform $u(t)$ - that brings $V(t)$ from V_r to V_{THR} at a constant *rate*, is the time-constant waveform $u(t)=k^*=(V_{THR}-V_r)/T$. For this example, $k^*=10\gg I_{ION}(V)$, which explains why $u^*(t)$ is that close to a rectangular waveform.

As a matter of fact, for very short stimulation times, the k^* tend to be high, while $I_{ION}(V)$ tends to be linear. Hence, the 'classic' rectangular (or square, SQR) waveform tends to also be close to energy-optimal.

Such facts are rather important as they lead us below (as evidence is accumulated) to a general form not only of $V^*(t)$, but also of $u^*(t)$.

Comparative properties the $V(t)$ growth profiles. The GE waveform may be an SQR waveform *in disguise*. I.e. some linear growth of the membrane voltage may still fit the one obtained upon ES with a GE. The motivation for this is in eqn. (36), where the first term vanishes with $T\gg\tau$.

Finally, the total electric charge conveyed by the ES source may have to be considered. For example, in the LM of eqn. (8) the total charge consists of a capacitive charge to raise the membrane voltage by a given amount (to V_{THR}), and resistive charge $\int_0^{T_{STIM}} V(t)/Rdt$. A similar situation occurs in the $MRG'02$ model due to the opposing axial currents.

So let us solve the following auxiliary problem:

Find a linear fit $\hat{V}(t)=\max[a(t-b),0]$ to the growing exponent $V(t)=(\mathbf{e}^{t/\tau}-1)/(\mathbf{e}^{T_{STIM}/\tau}-1)$, so that the ES source conveys the same resistive charge in the time interval $t\in[0,T_{STIM}]$. I.e. we want that:

$$\int_0^{T_{STIM}} \hat{V}(t)dt=\int_0^{T_{STIM}} V(t)dt=\tau-\frac{T_{STIM}}{\mathbf{e}^{T_{STIM}/\tau}-1}$$

Here, for simplicity (and without any loss of generality) we have assumed $V_r=0$ and $V_{THR}=1$.

For example with $\tau=T_{STIM}/4$, we obtain $b\approx 0.54\times T_{STIM}$, i.e. the linear-growth equivalent has more than twice shorter duration - e.g. with $T_{STIM}=5$, $b\approx 2.7$.

The latter result promotes intuition: with large opposing currents optimal ES cannot *afford* to last long. The transition of the membrane voltage from its rest to a threshold value is best performed rapidly. Hence, the shape of the $V(t)$ growth profile depend on the T_{STIM}/τ ratio. As seen, for $T_{STIM}\ll\tau$, the optimal $u(t)$ is close to rectangular, while with $T_{STIM}\gg\tau$, the GE is in effect equivalent to doing nothing for at least half of the duration, and then to a SQR waveform of at least doubled amplitude.

With quite similar reasoning, one can demonstrate that a 1st-order membrane voltage growth profile $V(t)=(1-\mathbf{e}^{t/\tau})/(1-\mathbf{e}^{T_{STIM}/\tau})$ in the time interval $t\in[0,T_{STIM}]$ is suboptimal and equivalent to linear growth, which has about twice longer duration.

Izhikevich model. Replacing $I_\Sigma(V^*)$ in eqn. (34) with the $I_{ION}(V)$ approximations from eqn. (14) or (15), see Box in Fig. 5:

$$C_m^2\ddot{V}=0.04^2(V-V_r)(V-V_{THR})[2V-(V_r+V_{THR})] \qquad (38)$$

As in the preceding model $V\equiv V^*$. Note that the dynamics of eqn. (38) has all FP's of $I_{ION}(V)$, as well as a third FP at $V=0.5(V_r+V_{THR})$, contributed by the derivative term $I'_{ION}(V)$.

Equation (38) can be solved analytically. However, it provides the solution in an implicit form and involves an incomplete elliptic integral of the first kind. Hence, we used the Matlab bvp4c BVP solver with boundary conditions $V^*(0)=V_r$ and $V^*(T_{STIM})=V_{THR}$.

Figure 5 illustrates the energy-optimal LAP solution $u^*(t)$ and the corresponding membrane voltage profile $V^*(t)$. The $I_{ION,0}(V)$ approximation of the ionic current is used for a case of very short duration ($T_{STIM}=10$ μs) and the $I_{ION,\infty}(V)$ approximation is used for a case of long duration ($T_{STIM}=5$ *ms*).

It is important to notice that - as with the LM model above, $u^*(t)\approx k^*+I_{ION}(V)$, where $k^*=(V_{THR}-V_r)/T$ (see the Box in Fig. 5).

According to eqns. (14) and (15) the opposing current in the IM can be presented in the general form:

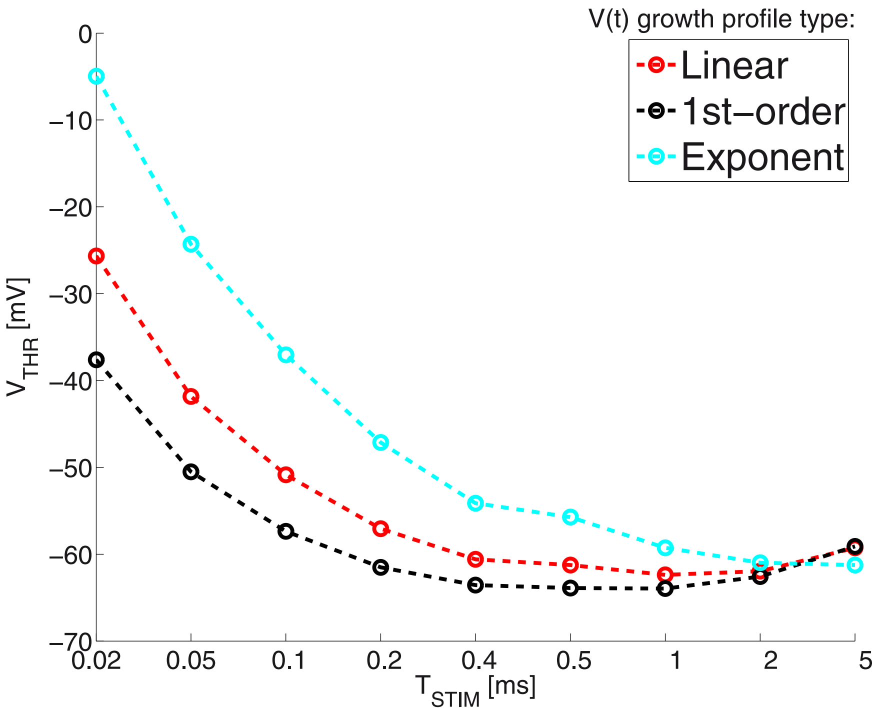

Figure 9. The actually computed V^*_{THR} as a function of T_{STIM} : Notice how the computed V_{THR} value is rather similar (almost matched) between the linear and exponential cases, for T_{STIM} respectively 2 and 5 ms; and between the 1st-order and linear cases, for T_{STIM} respectively 0.2 and 0.5 ms. see also Fig. 10.
doi:10.1371/journal.pone.0090480.g009

$$I_\Sigma(V^*) = gain \times \hat{I}_{ION}(V) \tag{39}$$

where the *nominal* $gain = 1$, and $\hat{I}_{ION}(V) = 0.04(V - V_r)(V - V_{THR})$.

To see how the optimal ES is affected by the level of opposing current, it is more than tempting to experiment with different *gain* values.

Hence, 3 *gain* cases are plotted in Fig. 5 - for the nominal *gain* (cyan traces) and two additional cases: the opposing current $I_\Sigma(V^*)$ is either doubled ($gain = 2$, red traces) or decreased two-fold ($gain = 1/2$, black traces). As could be intuitively expected from the general equation (24), when $I_{ION}(V) \to 0$ (very low ionic currents):

$$\int_0^{T_{STIM}} u^2 dt \sim \int_0^{T_{STIM}} \dot{V}^2 dt \tag{40}$$

By the *Cauchy-Schwartz inequality* in the space of continuous real functions, it is straightforward to show that the voltage trajectory $V^*(t)$ that minimizes eqn. (40) is such that $\dot{V}^*(t) = k^*$, where k^* is determined from the boundary conditions satisfied by $V^*(t)$. Hence:

Table 7. Minimal $V_{THR}(T_{STIM})[mV]$ values for the MRG'02 model, obtained for each $V(t)$ trajectory class.

T_{STIM}	Linear	1st-order	Exponent.
0.020	−25.649	−37.602	−4.963
0.050	−41.838	−50.515	−24.311
0.100	−50.852	−57.366	−37.032
0.200	−57.061	−61.506	−47.137
0.400	−60.588	−63.558	−54.124
0.500	−61.247	−63.889	−55.731
1.000	−62.378	−63.960	−59.255
2.000	−61.950	−62.578	−60.977
5.000	−59.273	−59.094	−61.249

doi:10.1371/journal.pone.0090480.t007

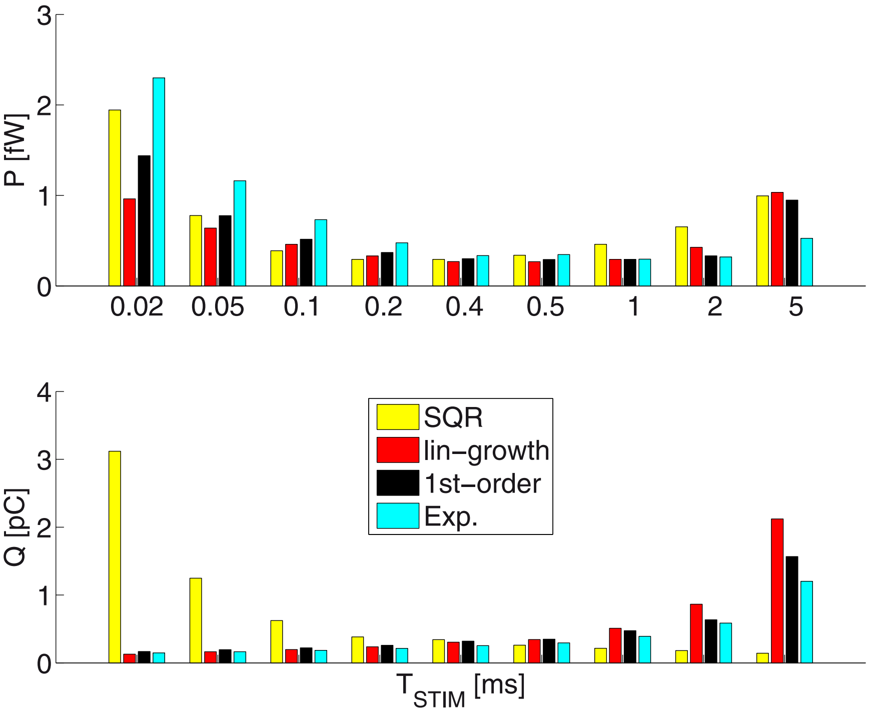

Figure 10. The energy P and charge-transfer Q values as a function of T_{STIM} : The linear-ramp voltage profile yields the best P performance for most of the durations. As in Fig. 8 notice that the P and Q values are quite similar for the linear and exponential cases, for T_{STIM} respectively 2 and 5 ms; and also for the 1st-order and linear cases, for T_{STIM} respectively 0.2 and 0.5 ms. Toward the P values electrode impedance of 1 $M\Omega$ is assumed. Contrasted: SQR stands for the square (or rectangular) stimulation waveform.
doi:10.1371/journal.pone.0090480.g010

$$k^* = \frac{V_{THR} - V_r}{T_{STIM}} \tag{41}$$

Just as in the preceding model, it is also $V^*(t)/V_{THR} \sim t/T_{STIM}$ with the shorter durations - which justifies the use of the resting approximation $I_{ION,0}(V)$.

HHM. Here the $I_\Sigma(V^*)$ of eqn. (34) is replaced with the resting-state - $I_{ION,0}(V)$, or asymptotic-state - $I_{ION,\infty}(V)$ ionic current approximations (see the Box in Fig. 6).

Toward $I_{ION,0}(V)$ the gate-state variables are factored out as follows: The fast state $m(t) \approx m_\infty(V)$, while the slower variables $h(t) \approx h_r = h_\infty(V_r)$, and $n(t) \approx n_r = n_\infty(V_r)$ are approximately at rest, assuming very short durations. Conversely, and assuming very long durations, toward $I_{ION,\infty}(V)$ all gate variables are approximately at their asymptotic value, corresponding to a given membrane voltage $V(t)$ (see Methods).

As with the IM, we used bvp4c to numerically solve the BVP of eqn. (34) with boundary conditions $V^*(0) = V_r$ and $V^*(T_{STIM}) = V_{THR}$.

Figure 6 follows a very similar format to Fig. 5.

Similarly to eqn. (39) above, $I_\Sigma(V)$ can also be assumed higher or lower. All the maximal ionic conductances in the HHM (see also Table 3) are temperature-dependent and are linearly proportional to the coefficient k_T:

$$k_T = Q_{10}^{(T - T_0)/10} \tag{42}$$

where $Q_{10} = 2.3$ and $T_0 = 23°$C. Hence with $T = 37°$C, according to eqn. (42) $k_T = 3.2094$. Let this be our *standard* case ($gain = 1$).

As we did with the IM, 3 gain cases are plotted in Fig. 6 for $I_\Sigma(V^*) = gain \times \hat{I}_{ION}(V)$. For the two additional cases the opposing current $I_\Sigma(V^*)$ is either doubled ($gain = 2$, red traces) or halved ($gain = 1/2$, black traces).

Once again - as with the LM and IM models above, $u^*(t) \approx k^* + I_{ION}(V)$ (see the Box in Fig. 6).

Numerical model simulation and optimal control. The IM was also evoked in the FHOC Methods section. It is therefore interesting to contrast the results of the LAP and FHOC approaches in identifying energy-optimal ES waveforms for the same ionic current model. For such comparison, the IM has the clear advantage of hiding no implementation specifics inside a *black box*.

The FHOC formalism (see Methods) is computationally efficient, but it is also subject to the similar limitations as most of the ad-hoc search approaches. Iterative numerical optimization requires an initial guess for the solution, and trying different starting arrays $u^{(0)}$ may alleviate a bit the propensity to converge to shallow local energy-minima.

Here it is also important to realize that in eqn. (16) the two terms to minimize in the $F(u)$ functional (a function of functions), namely the energy cost (17) and the penalty (18) may conflict each other. When the penalty gain $K_{penalty}$ in (18) is too low, the search will identify a lower-energy solution u, which however does not bring the membrane potential V_k up to the desired threshold value - i.e. $V_M \ll V_{THR}$. Conversely, a too high penalty gain $K_{penalty}$ will identify a very high-energy solution u, which is not only costly, but the membrane potential may also overshoot the threshold, since

Table 8. Minimal $Q(T_{STIM})[pico-Coulomb]$ values for the MRG'02 model, obtained for each $V(t)$ trajectory class.

T_{STIM}	SQR	Linear	1st-order	Exponent.
0.0200	3.1180	0.1279	0.1671	0.1467
0.0500	1.2472	0.1630	0.1946	0.1642
0.1000	0.6236	0.1959	0.2212	0.1847
0.2000	0.3832	0.2369	0.2583	0.2121
0.4000	0.3426	0.3045	0.3191	0.2545
0.5000	0.2605	0.3440	0.3492	0.2937
1.0000	0.2143	0.5093	0.4736	0.3910
2.0000	0.1808	0.8640	0.6361	0.5855
5.0000	0.1411	2.1216	1.5673	1.2018

doi:10.1371/journal.pone.0090480.t008

the 'getting there' is underestimated for the sake of the very last simulation steps.

As seen from Fig. 7 Panel B (which uses the $I_{ION,\infty}(V)$ approximation of the ionic current for the relatively long duration $T_{STIM}=2\ ms$), the linear growth profile is a reasonable estimate for the optimal membrane voltage profile $V^*(t)$. Hence:

$$u^*(t) \approx k^* + I_{ION,\infty}(V) \tag{43}$$

where k^* is given by eqn. (41). When $u^{(0)}$ is close to the LAP estimate $u^*(t)$ of eqn. (43), the FHOC iteration also consistently ends close to there (see Fig. 7, panel B). The cyan traces on Fig. 7 are the $u^*(t)$ and the resulting $V^*(t)$. With the LAP estimate, the FHOC approach resulted in a final membrane potential reasonably close to the desired threshold value - i.e. $V(T_{STIM}) = -50.106 \approx V_{THR} = -50$, even if the IM was simulated with the discretized LAP waveform $u^*(t)$ ($\Delta t = 10\ \mu s$).

The black traces illustrate the FHOC solution, computed for two different $u^{(0)}$ choices. For Panel A, $u^{(0)}$ was chosen to be all zeros. When all time-step entries $u^{(0)}$ were chosen to be equal to the upper bound $U=30$ (data not shown), due to the (discontinuous) AP event occurring mid-way the temporal horizon, the Matlab's fmincon solver remains *stuck* to the initially provided values.

Except for the case in Panel B, the $K_{penalty}$ meta-parameter had to be kept high ($K_{penalty}=70$) in order to respect the terminal constraint of $V(T_{STIM}) \approx V_{THR}$.

The total energy costs (all expressed as 2-norms of the obtained best $u(t)$) are respectively 161, 153.2 and 423.4 (for the discrete-time version) 186.7, 159.1 and 334.2 (for the continuous-time version).

Comparing these to $P(u^*) = 153.2$ (discrete-time) and $=157.4$ (continuous-time), the LAP-based solution is comparable to or superior than the FHOC solutions. The numerical FHOC solution on Fig. 7, panel A has converged to a local extremum. Note that a post-hoc correction (simple DC offset) is applied to the LAP-based estimate, which adjusts for the overshoot of V_{THR} when simulating the full (two-dimensional) IM. The overshoot is due to using the one-dimensional approximation, eqn. (15).

The results obtained here nicely illustrate multiple aspects of identifying energy-efficient waveforms through numerical model simulation and optimization. Clearly, pairing theoretical insights with numerical tools carries the best success potential.

Part I Results Summary

A number of more general observations on $u^*(t)$ can be made looking at the results this far.

Probably, the most significant result is that the use of LAP reduces the problem to the BVP, defined by eqn. (34), with $V^*(0) = V_r$ and $V^*(T_{STIM}) = V_{THR}$. We still need to have a very good idea of both $I_\Sigma(V)$ and V_{THR} to successfully solve for $V^*(t)$, and thence for $u^*(t)$, in a given particular situation.

We identify also the following key and practice-oriented optimality principles resulting from the LAP perspective.

1. The optimal sub-threshold membrane potential growth profile with relatively short durations T_{STIM} and low membrane conductivity:

   First, in all simple models we used up to here, the solution $V^*(t)$ of the ODE system, defined by eqn. (34), is quite close to a linear growth from $V^*(0) = V_r$ to $V^*(T_{STIM}) = V_{THR}$. Second, with the total current $I_\Sigma(V) \approx 0$ (e.g. low leak), then from eqn. (6), it follows that $u(t)$ will be exactly proportional to the rate of change of the membrane's potential $V(t)$. If $\dot{V}^*(t) \approx const$, then $u^*(t)$ is close to a SQR waveform.

2. The energy-efficient waveform depends directly on the temporal shape of currents at the AP initiation site.
3. The targeted V_{THR} membrane voltage threshold depends on stimulation duration, with a tendency to increase with T_{STIM}.
4. The exponential growth membrane voltage profiles $V(t)$ are equivalent to linear growths of shorter duration.

Part II - Multiple-compartment Model Results

Here we first extend the general (model-independent) LAP result of eqn. (34) to spatial-structure models (non-zero-dimensional, multi-compartment), which involve membrane-voltage distribution and propagation along cable structures.

LAP result generalization to multi-compartment models. There is a combinatorial explosion in both the number of parameters and the number of ways that multi-compartment models can be put together and used. Hence, there is much more than one way of generalizing the LAP result of eqn. (34).

Here we briefly present a variant, which appears to be one of the most straightforward generalizations.

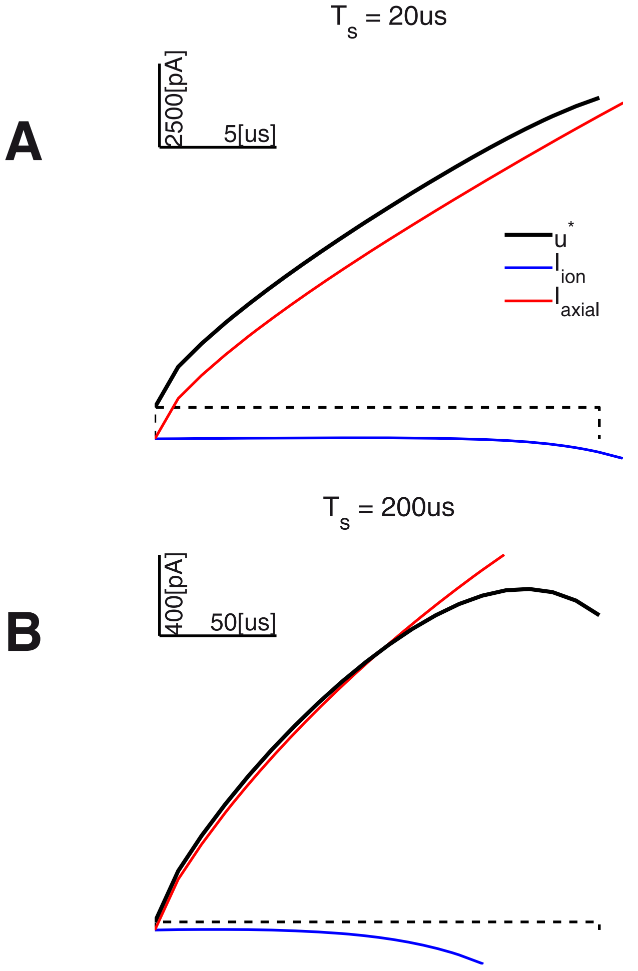

Figure 11. Optimal waveforms $u^*(t)$, $T_{STIM}=20$, 200 μs: The figure also provides the corresponding optimal SQR-like linear-growth-related current $C_m \times k^*$ (dashed black), as well as the components of I_Σ - respectively the I_{ION} (blue traces) and I_{axial} (red traces) current trajectories.
doi:10.1371/journal.pone.0090480.g011

With a multi-compartment model, eqn. (7) can be rewritten as:

$$\frac{\partial}{\partial t}V(t,Z)=u(t,Z)-I_\Sigma(V,Z) \qquad (44)$$

Without loss of generality, we used the variable Z to represent any 'spatial' model dimension. It could even stand for the compartment index in a discretized implementation.

Now, eqn. (7) is a partial DE, depending both on the temporal and the spatial model dimensions.

Assuming that we are free to manipulate $u(t,Z)$ in every compartment as we wish, the derivation sequence from eqn. (23) to eqn. (30) (see the LAP subsection in the Methods) still applies yielding a family of equations 'parameterized' by the location coordinate Z.

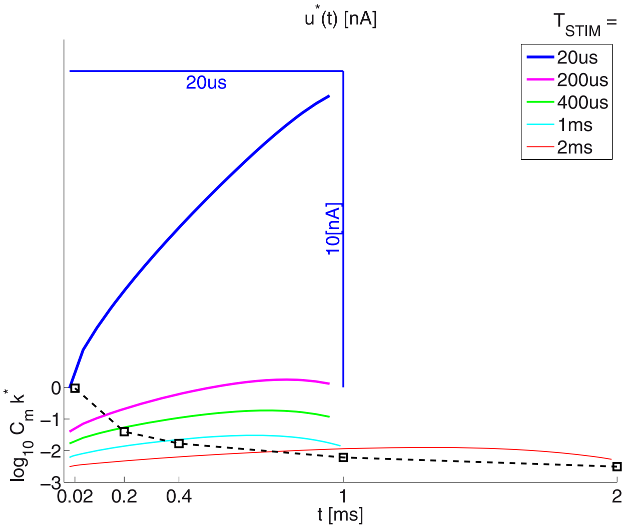

Figure 12. Optimal waveforms $u^*(t)$: see also Fig. 11. Notes: Since here $\forall t, \dot{V}^*(t)=k^*$, where k^* is given by eqn. (41), from eqn. (6) $u^*(t)=C_m k^*+I_\Sigma(V)$. The figure is optimized to present clearly both $u^*(t)$ and k^* **(*1)** The dashed trace at the bottom plots $\log_{10} C_m k^*$ as a function of T_{STIM} **(*2)** Toward equally good plot visibility, for all durations $T_{STIM}<1ms$, the waveforms $u^*(t)$ are *rubber-banded* to take the same graph width as the 1 ms-waveform. This is illustrated by the scale bars for the shortest duration $T_{STIM}=20\ \mu s$. **(*3)** The vertical scale is the same for all plots, except for the logarithmic offset, as defined by pt. (*1) above.
doi:10.1371/journal.pone.0090480.g012

Hence, we may obtain the generalization of eqn. (34) as:

$$C_m^2\frac{\partial^2}{\partial t^2}V^*(t,Z)=I_\Sigma(V^*,Z)\times\frac{\partial}{\partial V}I_\Sigma(V^*,Z) \qquad (45)$$

Like the extended eqn. (44), eqn. (45) is a partial DE, depending on both temporal and spatial boundary conditions. In particular, V_{THR} becomes a function of Z. It is no longer a single variable, but a whole spatial profile, subject to conditions such as the *safety factor* for propagation introduced in the cardiac literature [34].

The MRG'02 model: Toward upper bounds on $V_{THR}(T_{STIM})$. Multi-compartment models add complexity unseen with the single-compartment models. Wongsarnpigoon & Grill [8] used the peripheral-axon MRG'02 model [25] in a genetic-programming search for energy-efficient stimulation waveforms. The approach was somewhat similar to the FHOC described above. After thousands of iterations simulating the MRG'02 model, the identified waveforms were reminiscent of noisy truncated and vertically offset Gaussian's (Fig. 2 in [8]). In the light of analysis this far one might think that this reflects the shape of $I_\Sigma(V)$ for V ranging from the resting value (−80 mV) to some threshold V_{THR}.

In this work stimulation is assumed to be intracellular and at just one spatial location ($Z=0$, the center RN, see Methods) along the cable structure.

To suggest a version of optimal waveforms $u^*(t)$ for the MRG'02 model, we first estimate the membrane voltage threshold for each duration. One analytic way toward such estimates is

Table 9. Minimal $P(T_{STIM})[femto-Watt]$ values for the MRG'02 model, obtained for each $V(t)$ trajectory class.

T_{STIM}	SQR	Linear	1st-order	Exponent.
0.0200	1.9444	0.9620	1.4387	2.2993
0.0500	0.7778	0.6391	0.7765	1.1611
0.1000	0.3889	0.4596	0.5158	0.7325
0.2000	0.2937	0.3307	0.3692	0.4766
0.4000	0.2934	0.2693	0.3003	0.3352
0.5000	0.3392	0.2675	0.2913	0.3463
1.0000	0.4593	0.2934	0.2929	0.2954
2.0000	0.6535	0.4265	0.3321	0.3204
5.0000	0.9949	1.0339	0.9486	0.5263

doi:10.1371/journal.pone.0090480.t009

readily provided by the MRG'02 model. Recall also that with simpler models V_{THR} showed a tendency to increase with T_{STIM}.

Figure 8 presents a family of ionic current $I_{ION}(V,Z)$ approximations at the target site ($Z=0$), for a set of durations T_{STIM}. For each of the durations we assume that the membrane voltage trajectory $V(t)$ evolves according to a linear ramp from rest V_r to threshold V_{THR}. As the latter is unknown, we produced one such ramp for each V value on the horizontal (independent-variable) axis of the figure, and then computed the corresponding ionic current $I_{ION}(V)$ as described next.

Toward gross estimates of V_{THR}, we first solve approximately eqn. (10) for each gate-state:

$$\hat{x}(T)=x_0+(x_\infty(V)-x_0)(1-\mathbf{e}^{-T/\tau_x(\bar{V})}) \qquad (46)$$

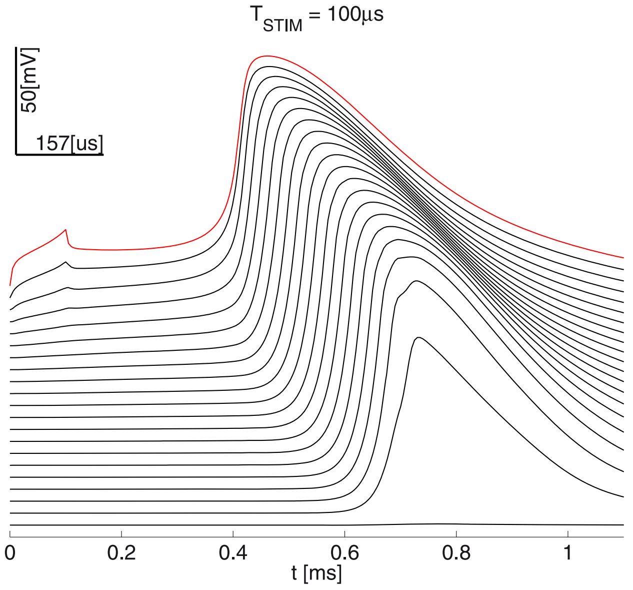

Figure 13. Propagating AP due to an optimal SQR (rectangular) waveform, $T_{STIM}=100$ μs: For the shortest durations, the plain rectangular waveform outperforms by P the ones associated to the linear-ramp voltage profile. One can see clearly that the steep rise of the SQR waveform yields an early superlinear ramping of the membrane voltage. However, the rectangular waveform requires a lot more charge Q to be transferred (see Fig. 10).
doi:10.1371/journal.pone.0090480.g013

where x_0 is the gate-state value at rest and $\bar{V}=(V_r+V)/2$ is the average excursion from the resting membrane voltage.

Figure 8 shows the obtained approximate ionic currents $I_{ION}(V(t))$ as a function of just V for three very different durations - $T_{STIM}=0.02$, 0.5 and 5 ms. For $T_{STIM}=5$ ms, the Box in the same figure illustrates the estimated proportions-to-rest $\hat{x}(T_{STIM}|V)/x_\infty(V_r)$ for each of the 4 gate-state variables, at the end of stimulation.

Why does such an analysis provide upper bounds on $V_{THR}(T_{STIM})$?

First, from the Box of Fig. 8 we can see that indeed the dynamics of the fast Na^+ ion channel subtype evolves before that of the other ion channels. Particularly, we see that the estimate for inactivating h gates suggests they are completely closed for $T_{STIM}=5$ ms and once V reaches around -40 mV.

On the other hand from the main Fig. 8, one can see that this analysis gives the intervals $V\in[V_{rest},V_{UB}]$ in which the approximate ionic currents $I_{ION}(V)<0$ (i.e. remain *depolarizing*).

Clearly if $V_{THR}(T_{STIM})$ is not reasonably within $[V_{rest},V_{UB}]$, no miracle would yield an AP at the target location, since I_{ION} becomes *repolarizing* outside of these bounds.

Interestingly, the analysis also predicts *lowering* of V_{THR} with longer durations. This result is exactly the opposite of what was observed with the simpler models of the HH-type, where I_{ION} was *repolarizing* for $V\in[V_{rest},V_{THR}]$.

The numerical experiments we conducted were fully consistent with the above predictions, and some upper bounds were also quite tight.

The MRG'02 model: numerical experiments. We conducted four series of numerical experiments in search of the optimal waveforms $u^*(t)$ for the MRG'02 model. Each series was computed for the same set of 9 durations $T_{STIM}=20$, 50, 100, 200, 400 and 500 μs; 1, 2 and 5 ms (for the sake of better visibility, only the most representative subsets are illustrated in full detail).

The four series differed by the chosen voltage-clamp temporal growth profile $V(t,0)$ at the targeted RN location and A baseline series involved finding the threshold rectangular stimulation amplitude. In all series, the constraint was to observe a propagating AP at the latest within 1 ms after the end of stimulation.

With $\Delta V=V_{THR}(T_{STIM})-V_r$, where the minimum V_{THR} was found (with 0.001 mV tolerance) using the same type of golden-section search algorithm as per the optimal SQR amplitude.

And the three LAP-driven series were:

linear growth

$$V(t) = V_r + \Delta V t / T_{STIM} \tag{47}$$

exponential growth

$$V(t) = V_r + \Delta V(\mathrm{e}^{t/\tau} - 1)/(\mathrm{e}^{T_{STIM}/\tau} - 1) \tag{48}$$

1-st order growth

$$V(t) = V_r + \Delta V(1 - \mathrm{e}^{-t/\tau})/(1 - \mathrm{e}^{-T_{STIM}/\tau}) \tag{49}$$

The corresponding $u(t,0)$ ES waveforms were computed from eqn. (44) with $Z=0$.

The MRG'02 model: numerical results. Figure 9 and table 7 illustrate the obtained V_{THR} as a function of T_{STIM}.

The computed optimal values of V_{THR} are often similar for two adjacent durations either between the linear and 1-st order, or between the linear and exponential growth (EG). 1-st order is usually similar to its right-hand linear neighbor (for the next *longer* duration). Conversely, EG is similar to its left-hand linear neighbor (for the previous *shorter* duration).

This is consistent with and best interpreted in the light of our growth-profiles comparison (see the dedicated subsection on page 13). There we saw that indeed an EG $V(t)$ trajectory is approximately equivalent to linear growth of about twice shorter duration. As for 1-st order growth, clamping the voltage to its plateau will tend to be similar to a linear growth of about twice longer duration. Recall also that 1-st order is the 'reverse-time' analog of EG.

Figure 10 and tables 7, 8 illustrate the obtained optimal-waveforms' energy P and charge-transfer Q values as a function of T_{STIM}.

The linear-growth strategy is the one that tends to perform best across the board, except for the 2 longest durations, and as predicted by the comparative (linear vs exponential growth) analysis, based on the 0D LM.

Figure 3 illustrates the propagating AP's, corresponding to the two representative linear and exponential voltage-clamp temporal growth profiles at the stimulation site $V(t,0)$. The figure also shows the spatial profiles of the membrane voltage and intracellular potential at the end of stimulation for the two growth cases.

Consistently with the analysis in the subsection on the comparative properties of the $V(t)$ growth profiles, we found out that the spatial distributions of membrane voltage and intracellular potentials at the end of stimulation were reasonably similar - e.g. between the optimal linear growth voltage-clamp for $T_{STIM}=2$ *ms*, Fig. 3 (Panels A, C) and the optimal exponential growth with $T_{STIM}=5$ *ms*, Fig. 3 (Panels B, D).

Note that we expect from an approximately globally optimal stimulation waveform $u^*(t)$ to yield a *specific* distribution of membrane voltages $V(T_{STIM},Z)$ at the end of the stimulation. We call this distribution tentatively the *invariant* spatial profile of the membrane voltage. Importantly, such a profile will differ for any different duration T_{STIM} *even* when the corresponding waveform $u^*(t)$ is globally optimal. This is due for example to the small spatial constant λ, which controls the spatial diffusion with time.

However, if the spatial profile is about the same for different durations T_{STIM} and the corresponding different waveforms $u^*(t)$ (see Panels B and D in Fig. 3), then both waveforms may be optimal. Recall that linear fits to both the optimal 1-st order growth and the optimal exponential growth with durations $T_{STIM}=5$ *ms* have duration $\approx 0.46 \times T_{STIM} = 2.3$ *ms*. Thus, all of the above cases may yield quasi-invariant spatial potentials at the end of stimulation, and may also be otherwise similar.

For two representative linear-growth cases Fig. 11 illustrates the corresponding waveforms $u^*(t)$ and their construction in detail.

Finally, Fig. 12 uses the same-vertical-scale to compare the relative contributions of the growth rate and the compensated re-polarizing node currents for each different duration. The waveforms' offsets (due to k^*) are inversely proportional to duration. This readily compares qualitatively with the results in [8]. Especially for very short durations (e.g. $T_{STIM}=20\mu s$), the optimal waveform $u^*(t)$ has a significant rectangular component (see also the optimality-analysis for the simple 0D models). Further parallels may be made for the relatively shorter durations ($\leq 200\mu s$).

Numerous essential differences in the approach preclude further objective comparisons. Interestingly however, for the longer durations ($T \geq 0.5$ *ms*) the results in [8] show very little (if any) variation with T_{STIM} (there called pulse-width, PW).

Finally, with long PW's in [8] most of the stimulation's energy is delivered toward the middle of the active period. This late and peaky delivery requires additional analysis and comparisons of the actually achieved waveform-energy levels, which cannot be done in its details at this time. However, we return to the late delivery policy in the Discussion (see below), where it is deemed equivalent to a shorter-duration case.

The latter provides a clue why such significant delivery differences would not be at odds with the very narrow 95% confidence intervals that resulted from the genetic algorithm in [8], and seeming to preclude different optimal waveforms.

Discussion and Conclusions

In eqn. (23), we addressed *directly* the electric power required for driving the excitable-tissue membrane potential $V(t)$ from its resting (V_r) to its threshold value (V_{THR}) through a stimulation of *fixed* duration. Through the LAP perspective, we obtained eqn. (34) - a general (model-independent) description of the energy-optimal time-course of the excitable-tissue's membrane potential $V^*(t)$.

We would like to bring the reader's attention to three specific conclusions.

The first is related to the intuition gained with respect to the evolution of the membrane potential $V^*(t)$. This optimality principle is best demonstrated by the simplest linear sub-threshold model (LM). Let ES circumstances be characterized by large opposing currents (e.g. the leak LM current) over long durations. This situation is physically analogous to filling with water a bucket which has large holes in its bottom. Since only the final outcome is important (i.e. we want the bucket full at the final time T), the best policy is to do nothing for most of the duration and then be able to dump a very large amount of water in the bucket over very short time. From experience, we know that works for even an unplugged sink. Moreover, we saw that the same intuition transfers to more refined models (e.g. the HHM or the MRG'02) as do nothing for most of the duration means that we are still around the resting V and hence there is no danger of Na^+ ionic-channel deactivation.

The second take-home message is that the use of LAP principles *jointly* with numerical approaches (e.g. the classical FHOC) provides a mathematically sound and practical waveform optimi-

zation approach, providing more assurance toward the quality of the final outcome.

And finally, a note of humility is in perfect order. In this work we just slightly opened the door to using the LAP ideas for optimal ES. There are many more aspects to tackle than the ones that we can address in this short paper as 'proof of concept'. In particular we would like to extend the method for extracellular stimulation in forthcoming work. The motivation for doing so is at least twofold. On the one hand, extracellular stimulation has far more practical relevance. On the other hand, the only way we could rigorously employ the general LAP solution of eqn. (45) is to consider a model where we are free to manipulate $u(t,Z)$ in every compartment or at every spatial location.

A direction for such manipulation is provided by the *activating function* concept [15,20,25], which supplies every compartment with a virtual injected current. In the context of extracellular stimulation, we will also have to properly address the conditions for stable AP propagation (see [15,35] for an extensive treatment of the subject). The optimal pattern of extracellular potentials (size of depolarized and hyperpolarized regions) depends on the distance to the electrode. These conditions would also naturally provide the spatial voltage profile at the end of the stimulation, needed to properly solve the PDE of eqn. (45).

Here we took a shortcut path by assuming that intuitions gained with single-compartment models suffice. This may be partially true with the specific MRG'02 setup that we addressed, but does not hold in general. Hence, the LAP results are *approximate*. A clue is provided by the slightly lower P values of the optimal rectangular waveform, for $T_{STIM} = 100$ and 200 μs - see Table 9. As can be seen from Fig. 9, no benefit in terms of lower V_{THR} can be associated to the steep rise of the rectangular waveform, since V_{THR} is expected to be higher, esp. for dramatically shorter durations. This was further confirmed by numerical testing with dual linear (high/low rate) $V(t)$ rise schedules (data not shown), which all had inferior performance to the baseline simple linear-growth protocol. However, the rectangular waveform also leads to steep capacitive decay of $V(t)$ at the end of the stimulation, which may trigger specific patterns of additional depolarizing currents.

For the shortest durations, the plain rectangular waveform outperforms by P the ones associated to the linear-ramp voltage profile (see Fig. 10). On Fig. 13 one can see that the steep rise of the SQR waveform yields an early *super-linear* ramping of the membrane voltage. However, the rectangular waveform requires a lot more charge Q to be transferred.

In practical situations many more additional aspects need to be addressed. E.g. stimulation needs to be charge balanced. This is a necessity for implanted devices and also debatably important for transcutaneous applications. Such stimulation will have an effect on the optimal threshold intensity of the cathodic pulse [36]. One would expect that a pre- or post- anodic pulse would also have a significant effect on the optimal waveform. Moreover, its own shape would be subject to optimization - e.g. to minimize the overall energy level required - a cost suitable for the design of implanted devices.

We hope that the analysis and numerical evidence provided in this work may convince the reader of the practical benefits of applying the LAP principles toward the design of energy-efficient ES.

Author Contributions

Conceived and designed the experiments: NIK SMD AV. Performed the experiments: NIK. Analyzed the data: NIK SMD AV FR. Contributed reagents/materials/analysis tools: NIK SMD AV. Wrote the paper: NIK SMD AV FR MS.

References

1. Liberson W, Holmquest M, Scot D, Dow M (1961) Functional electrotherapy: stimulation of the peroneal nerve synchronized with the swing phase of gait of hemiplegic patients. Arch Phys Med Rehabil 42: 101–105.
2. Dhillon G, Horch K (2005) Direct neural sensory feedback and control of a prosthetic arm. IEEE Trans Neural Syst Rehab Eng 13(4): 468–72.
3. Fetz EE (2007) Volitional control of neural activity: implications for brain-computer interfaces. Journal of Physiology-London 579: 571–579.
4. Doherty J, Lebedev M, Hanson T, Fitzsimmons N, Nicolelis M (2009) A brain-machine interface instructed by direct intracortical microstimulation. Front Integr Neurosci 3: 20.
5. Lucas T, Fetz E (2013) Myo-cortical crossed feedback reorganizes primate motor cortex output. J Neurosci 33(12): 5261–74.
6. Sahin M, Tie Y (2007) Non-rectangular waveforms for neural stimulation with practical electrodes. J Neural Eng 4(3): 227–33.
7. Foutz T, McIntyre CC (2010) Evaluation of novel stimulus waveforms for deep brain stimulation. Journal of Neural Engineering 7: 066008.
8. Wongsarnpigoon A, Grill WM (2010) Energy-efficient waveform shapes for neural stimulation revealed with a genetic algorithm. Journal of Neural Engineering 7(4): 046009.
9. Wongsarnpigoon A,Woock JP, Grill WM (2010) Efficiency analysis of waveform shape for electrical excitation of nerve fibers. IEEE Trans Neural Syst Rehab Eng 18: 319–328.
10. Weiss G (1901) Sur la possibilite de rendre comparable entre eux les appareils servant a l'excitation electrique. ArchItalBiol 35: 413–446.
11. Lapicque L (1907) Recherches quantitatives sur l'excitation electrique des nerfs traites comme une polarization. J Physiol (Paris) 9: 622.
12. Lapicque L (1926) L'excitabilite en Fonction du Temps. La Chronaxie, sa Signification et sa Mesure. Presses Universitaries de France, Paris.
13. Lapicque L (1931) Has the muscular substance a longer chronaxie than the nervous substance? The Journal of Physiology 73: 189–214.
14. Lykknes A, Opitz DL, Tiggelen BV, editors (2012) For Better or For Worse? Collaborative Couples in the Sciences, volume 44 of Science Networks. Historical Studies. Basel: Springer-Birkhaeuser, 319 pp.
15. Rattay F (1990) Electrical Nerve Stimulation: Theory, Experiments and Applications. Wien, NY: Springer-Verlag.
16. Geddes LA (2004) Accuracy limitations of chronaxie values. IEEE Trans Biomed Eng 51: 176–181.
17. Feynman R, Leighton R, Sands M (1964) The principle of least action. IN: The Feynman Lectures on Physics Addison-Wesley, vol.II, ch.19.
18. Rall W (1977) Core conductor theory and cable properties of neurons. In: Handbook of Physiology - The Nervous System (APS) vol 1, chap 3, 39–97.
19. Katz B (1937) Experimental evidence for a non-conducted response of nerve to subthreshold stimulation. ProcRoySocLondon serB 124: 244–276.
20. Rattay F, Wenger C (2010) Which elements of the mammalian central nervous system are excited by low current stimulation with microelectrodes? Neuroscience 170: 399–407.
21. Hu W, Tian C, Li T, Yang M, Hou H, et al. (2009) Distinct contributions of $Na_{v1:6}$ and $Na_{v1:2}$ in action potential initiation and backpropagation. Nat Neurosci 12: 996–1002.
22. Izhikevich E (2007) Dynamical Systems in Neuroscience. Cambridge, MA: MIT Press.
23. Fitzhugh R (1961) Impulses and physiological states in theoretical models of nerve membrane. Biophys J 1: 445–66.
24. Izhikevich E (2004) Which model to use for cortical spiking neurons? IEEE Transactions on Neural Networks 15(5).
25. McIntyre CC, Richardson AG, Grill WM (2002) Modeling the excitability of mammalian nerve fibers: Inuence of afterpotentials on the recovery cycle. Journal of Neurophysiology 87: 995–1006.
26. Ranck JJB (1975) Which elements are excited in electrical stimulation of mammalian central nervous system: A review. Brain Research 98: 417–440.
27. Rattay F (1998) Analysis of the electrical excitation of cns neurons. IEEE Trans Biomed Eng 45: 766–772.
28. Rattay F (1999) The basic mechanism for the electrical stimulation of the nervous system. Neuroscience 89: 335–46.
29. Danner S (2010) Master thesis: Computer simulation of electrically stimulated nerve fibers in the human spinal cord. TU Vienna, Inst for Scientific Computing & Analysis, Advisor: F Rattay.
30. Danner SM, Hofstoetter US, Ladenbauer J, Rattay F, Minassian K (2011) Can the human lumbar posterior columns be stimulated by transcutaneous spinal cord stimulation? A modeling study. Artificial Organs 35: 257–262.
31. Grill W (2010). Personal comm. w.r.t. possible printing errors in the MRG'02 model's original 2002 publication.
32. Sage AP, Melsa JL (1971) System Identification, volume 80 of Mathematics in Science and Engi-neering. Academic Press, 221 pp.

33. Jezernik S, Morari M (2005) Energy-optimal electrical excitation of nerve fibers. IEEE Trans Biomed Eng 52(4): 740–3.
34. Shaw RM, Rudy Y (1997) Ionic mechanisms of propagation in cardiac tissue. roles of the sodium and l-type calcium currents during reduced excitability and decreased gap junction coupling. Circ Res 81: 727–41.
35. Rattay F, Paredes LP, Leao RN (2012) Strength-duration relationship for intra- versus extracellular stimulation with microelectrodes. Neuroscience 214: 1–13.
36. Hofmann L, Ebert M, Tass PA, Hauptmann C (2011) Modified pulse shapes for effective neural stimulation. Frontiers Neuroeng 4: 9.
